# Association of Alzheimer Disease With Life Expectancy in People With Down Syndrome

**DOI:** 10.1001/jamanetworkopen.2022.12910

**Published:** 2022-05-23

**Authors:** Maria Florencia Iulita, Diana Garzón Chavez, Maria Klitgaard Christensen, Natalia Valle Tamayo, Oleguer Plana-Ripoll, Sonja A. Rasmussen, Marta Roqué Figuls, Daniel Alcolea, Laura Videla, Isabel Barroeta, Bessy Benejam, Miren Altuna, Concepción Padilla, Jordi Pegueroles, Susana Fernandez, Olivia Belbin, María Carmona-Iragui, Rafael Blesa, Alberto Lleó, Alexandre Bejanin, Juan Fortea

**Affiliations:** 1 Sant Pau Memory Unit, Department of Neurology, Hospital de la Santa Creu i Sant Pau, Biomedical Research Institute Sant Pau, Universitat Autònoma de Barcelona, Barcelona, Spain; 2 Center of Biomedical Investigation Network for Neurodegenerative Diseases, Madrid, Spain; 3 National Centre for Register-based Research, Aarhus University, Aarhus, Denmark; 4 Departments of Pediatrics and Obstetrics and Gynecology, University of Florida College of Medicine, Gainesville, Florida; 5 Department of Epidemiology, University of Florida College of Public Health and Health Professions and College of Medicine, Gainesville, Florida; 6 Iberoamerican Cochrane Centre, Biomedical Research Institute Sant Pau, Barcelona, Spain; 7 Barcelona Down Medical Center, Fundació Catalana Síndrome de Down, Barcelona, Spain

## Abstract

**Importance:**

People with Down syndrome have a high risk of developing Alzheimer disease dementia. However, penetrance and age at onset are considered variable, and the association of this disease with life expectancy remains unclear because of underreporting in death certificates.

**Objective:**

To assess whether the variability in symptom onset of Alzheimer disease in Down syndrome is similar to autosomal dominant Alzheimer disease and to assess its association with mortality.

**Design, Setting, and Participants:**

This study combines a meta-analysis with the assessment of mortality data from US death certificates (n = 77 347 case records with a *International Classification of Diseases* code for Down syndrome between 1968 to 2019; 37 900 [49%] female) and from a longitudinal cohort study (n = 889 individuals; 46% female; 3.2 [2.1] years of follow-up) from the Down Alzheimer Barcelona Neuroimaging Initiative (DABNI).

**Main Outcomes and Measures:**

A meta-analysis was conducted to investigate the age at onset, age at death, and duration of Alzheimer disease dementia in Down syndrome. PubMed/Medline, Embase, Web of Science, and CINAHL were searched for research reports, and OpenGray was used for gray literature. Studies with data about the age at onset or diagnosis, age at death, and disease duration were included. Pooled estimates with corresponding 95% CIs were calculated using random-effects meta-analysis. The variability in disease onset was compared with that of autosomal dominant Alzheimer disease. Based on these estimates, a hypothetical distribution of age at death was constructed, assuming fully penetrant Alzheimer disease. These results were compared with real-world mortality data.

**Results:**

In this meta-analysis, the estimate of age at onset was 53.8 years (95% CI, 53.1-54.5 years; n = 2695); the estimate of age at death, 58.4 years (95% CI, 57.2-59.7 years; n = 324); and the estimate of disease duration, 4.6 years (95% CI, 3.7-5.5 years; n = 226). Coefficients of variation and 95% prediction intervals of age at onset were comparable with those reported in autosomal dominant Alzheimer disease. US mortality data revealed an increase in life expectancy in Down syndrome (median [IQR], 1 [0.3-16] years in 1968 to 57 [49-61] years in 2019), but with clear ceiling effects in the highest percentiles of age at death in the last decades (90th percentile: 1990, age 63 years; 2019, age 65 years). The mortality data matched the limits projected by a distribution assuming fully penetrant Alzheimer disease in up to 80% of deaths (corresponding to the highest percentiles). This contrasts with dementia mentioned in 30% of death certificates but is in agreement with the mortality data in DABNI (78.9%). Important racial disparities persisted in 2019, being more pronounced in the lower percentiles (10th percentile: Black individuals, 1 year; White individuals, 30 years) than in the higher percentiles (90th percentile: Black individuals, 64 years; White individuals, 66 years).

**Conclusions and Relevance:**

These findings suggest that the mortality data and the consistent age at onset were compatible with fully penetrant Alzheimer disease. Lifespan in persons with Down syndrome will not increase until disease-modifying treatments for Alzheimer disease are available.

## Key Points

**Question:**

Is age at onset of Alzheimer disease in people with Down syndrome as consistent as in autosomal dominant forms, and is the association of the disease with mortality compatible with near full penetrance?

**Findings:**

In this meta-analysis and cohort study, the variability of age at symptom onset in Down syndrome was comparable to autosomal dominant Alzheimer disease. The mortality data and the consistent age at onset were compatible with fully penetrant Alzheimer disease.

**Meaning:**

These findings suggest that closing the life expectancy gap for individuals with Down syndrome compared with the general population will require effective prevention or management of Alzheimer disease.

## Teaser

This meta-analysis assesses whether the variability in symptom onset of Alzheimer disease in Down syndrome is similar to autosomal dominant Alzheimer disease and its association with mortality.

## Introduction

Down syndrome is the most frequent genetic cause of intellectual disability due to the presence of all or part of a third copy of chromosome 21, which occurs approximately in 1:800 births. In Europe and the US, current estimates indicate a population prevalence of 5.6 and 6.7 per 10 000 individuals, respectively, which translates to more than 200 000 people in the US^1^ and more than 400 000 people in Europe.^2^

Down syndrome is a complex condition with multiple associated comorbidities that vary throughout the lifespan.^3^ Improvements in health care, most notably surgery for congenital heart defects, have remarkably increased life expectancy, from approximately 5 years in the 1950s to roughly 60 years in the 2020s.^4,5^ However, this still represents a survival difference of about 20 years when compared with the general population.

A consequence of this increased life expectancy has been the emergence of age-related diseases, most importantly Alzheimer disease, which has a risk of more than 90% by the seventh decade for people with Down syndrome.^6^ The strong association between Alzheimer disease and Down syndrome has a genetic basis through a gene-dose effect of the amyloid precursor protein (*APP*) gene located on chromosome 21, which is triplicated in this population. While the triplication of other genes on chromosome 21 can also contribute to amyloid aggregation,^7^ *APP* triplication is necessary and sufficient to cause dementia.^8^ Consequently, neuropathologic changes associated with Alzheimer disease, namely amyloid-β (Aß) plaques and neurofibrillary tangles composed of hyperphosphorylated tau, are universally seen in persons with Down syndrome by age 40 years,^9^ and biomarker changes follow a predictable course that is strikingly similar to that found in autosomal dominant Alzheimer disease.^10^ The latter represents genetic cases, which are caused by fully penetrant mutations in the *APP*, *Presenilin 1 (PSEN1)*, or *Presenilin 2 (PSEN2)* genes, leading to early-onset dementia at a predictable age, typically between age 30 and 60 years, depending on the mutation.

Because of these similarities, Down syndrome has been conceptualized as genetically determined Alzheimer disease, just like the autosomal dominant forms.^11,12^ However, the implications of this conceptualization on the predictability of symptom onset in Down syndrome and the limit Alzheimer disease imposes on life expectancy, have not been fully explored. Indeed, many have emphasized the variability in age at onset of dementia in Down syndrome,^13,14,15,16,17,18,19^ in sharp contrast with the recognized predictability of symptom onset in autosomal dominant forms.^20^ As such, constructs like the estimated years to symptom onset (EYO), which have been developed and applied with success to study the natural history of Alzheimer disease in autosomal dominant forms,^21^ have been less used in Down syndrome and remain controversial. This view represents an important setback for the inclusion of adults with Down syndrome in preventive trials, and for the development of appropriate care guidelines for patients and families.

For these purposes, we conducted a meta-analysis to determine the age at onset, age at death, and duration of Alzheimer disease dementia in adults with Down syndrome and compared the variability in disease onset with that reported in autosomal dominant Alzheimer disease. Based on these estimates, we constructed a hypothetical distribution of age at death in people with Down syndrome, under the assumption that Alzheimer disease had full penetrance and was lethal. We compared these results with mortality data from people with Down syndrome obtained from US death certificates in the past 50 years. We also investigated the underreporting of dementia in death certificates by imputing data based on the hypothetical distribution and the US Centers for Disease Control and Prevention (CDC) data and collecting mortality data from the Down Alzheimer Barcelona Neuroimaging Initiative (DABNI) clinical cohort.^10^

## Methods

### Systematic Review and Meta-analysis

This meta-analysis adhered to the Preferred Reporting Items for Systematic Reviews and Meta-analyses (PRISMA) reporting guideline. The review protocol and search strategy were registered in the International Prospective Register of Systematic Reviews (CRD42020203967). We conducted a systematic search to examine the age at onset, age at death, and duration of Alzheimer disease dementia among persons with Down syndrome using the following databases: PubMed/Medline, Embase, Web of Science, and CINAHL (eTable 1 in the Supplement). OpenGray was used for gray literature. Further details can be found in the eMethods in the Supplement.

#### Eligibility Criteria and Data Extraction

Studies were considered if they contained numerical results of the following outcomes among persons with Down syndrome: (1) age at onset or age at diagnosis of Alzheimer disease dementia, defined via sufficiently explained methodological criteria and based on expert clinical judgment or dementia criteria; (2) age at death in those with Alzheimer disease; or (3) disease duration (defined as the time from dementia onset/diagnosis to death). We imported records into the Covidence software version 2640 to assist the screening process. Two independent reviewers (MFI and DGC) screened all titles and abstracts to identify studies for full-text review. The risk of bias was assessed using a quality assessment tool (eTable 2 in the Supplement) adapted from the scale of McGrath et al.^22^ Further details are included in the eMethods in the Supplement.

#### Data Synthesis and Analysis

Pooled estimates with corresponding 95% CIs were calculated using random-effects meta-analysis with the DerSimonian–Laird method.^23^ Analyses were done in Stata version 15.1 using the metan package version 3.03 (StataCorp). Heterogeneity was assessed with the *I^2^* index.^24^ Further details on the subgroup analyses are in eMethods in the Supplement.

To compare the variability in age at onset of Alzheimer disease in Down syndrome with that of autosomal dominant forms, we used 2 approaches: (1) computing the coefficients of variation of age at onset for each study individually (SD/mean × 100), and (2) calculating the time interval during which 95% of the individuals will develop cognitive symptoms (prediction intervals [95% PI], calculated as mean ± 1.96 × SD). For Down syndrome, these values were calculated from our systematic review of 44 studies on Alzheimer disease dementia. For autosomal dominant Alzheimer disease, these values were calculated from 60 studies (with n ≥ 5) with data on age at symptom onset available in a previously published study.^20^ Data collection took place from August 2020 to February 2021, and data analysis took place from February 2021 to July 2021.

### Mortality Analyses

#### Hypothetical Death Distribution Assuming Full Penetrance of Alzheimer Disease

Data analysis was performed in R version 4.0.4 (R Project for Statistical Computing). We used pooled data from the systematic review calculated with a fixed-effect model^25^ to describe a hypothetical normal distribution of mortality in Down syndrome, assuming fully penetrant Alzheimer disease. The hypothetical death distribution was obtained from the sum of 2 independent distributions (age at onset and duration) as follows: mean_death_ = mean_onset_ + mean_duration_ and SD_death_ = sqrt (SD_onset_^2^ + SD_duration_^2^). We also examined the distribution using the estimate of age at death in those with Alzheimer disease.

#### US Mortality Data

We contrasted data obtained from the hypothetical death distribution with data obtained from Multiple-Cause Mortality Files from 1968 to 2019, compiled by the CDC. We used the following *International Classification of Diseases *(*ICD*) codes to select records from people with Down syndrome: 759.3 (*ICD*, *Eighth Revision*: 1968-1997), 758.0 (*ICD*, *Ninth Revision*: 1979-1998), and Q90 *ICD*, *Tenth Revision*: 1999-2019). Further details are in eMethods in the Supplement. We calculated the median age at death and the 10th, 25th, 75th, and 90th percentiles by year.

We next examined the trend in the reporting of congenital heart disease and dementia in people with Down syndrome between 1968 to 2019 (eTable 3 in the Supplement). Given that dementia is typically underreported on death certificates,^26,27^ we performed an exploratory analysis to compare the real age at death (CDC data) with the estimated ages at death in the fully penetrant hypothetical distribution by calculating the difference between the 2 for each percentile. Whenever the difference was close to 0, we assumed that Alzheimer disease could be the contributor. When this difference was larger, we attributed it to other causes of death. We plotted this difference for each percentile, and we visually assessed the graph and mathematically tested if there was an inflection point by implementing threshold regressions using the R library “chngpt”.

#### Mortality Data From the DABNI Cohort

We collected mortality data from DABNI, a single-center prospective cohort that is part of a public health plan developed for the screening of Alzheimer disease in adults with Down syndrome in Catalonia, Spain, through clinical and multimodal biomarker assessments.^10^ The sample is representative of the adult population aged 18 years or older with Down syndrome in Catalonia, Spain. All participants or their legal representatives were required to give informed consent. The Clinical Research Ethics Committee at Hospital Sant Pau reviewed and approved all DABNI protocols. Between February 1, 2013, and July 31, 2021, we evaluated 889 individuals with Down syndrome (46% females), with a mean follow-up of 3.2 (2.1) years. Details about the Alzheimer diagnostic procedures are in eMethods in the Supplement.

## Results

### Systematic Review and Meta-analysis

We identified 7004 studies for title and abstract screening after the removal of duplicates (eFigure 1 in the Supplement). Of these, 261 underwent full-text review, and 52 (20%) met the inclusion criteria. Outcomes of interest were extracted from studies from 8 different countries. No time restriction was imposed in the search, and eligible studies were published between 1985 and 2020. A description of included studies is available in eMethods and eTable 4 in the Supplement, and the studies are cited in the reference list.^6,10,28,29,30,31,32,33,34,35,36,37,38,39,40,41,42,43,44,45,46,47,48,49,50,51,52,53,54,55,56,57,58,59,60,61,62,63,64,65,66,67,68,69,70,71,72,73,74,75,76,77^

**Figure 1.**
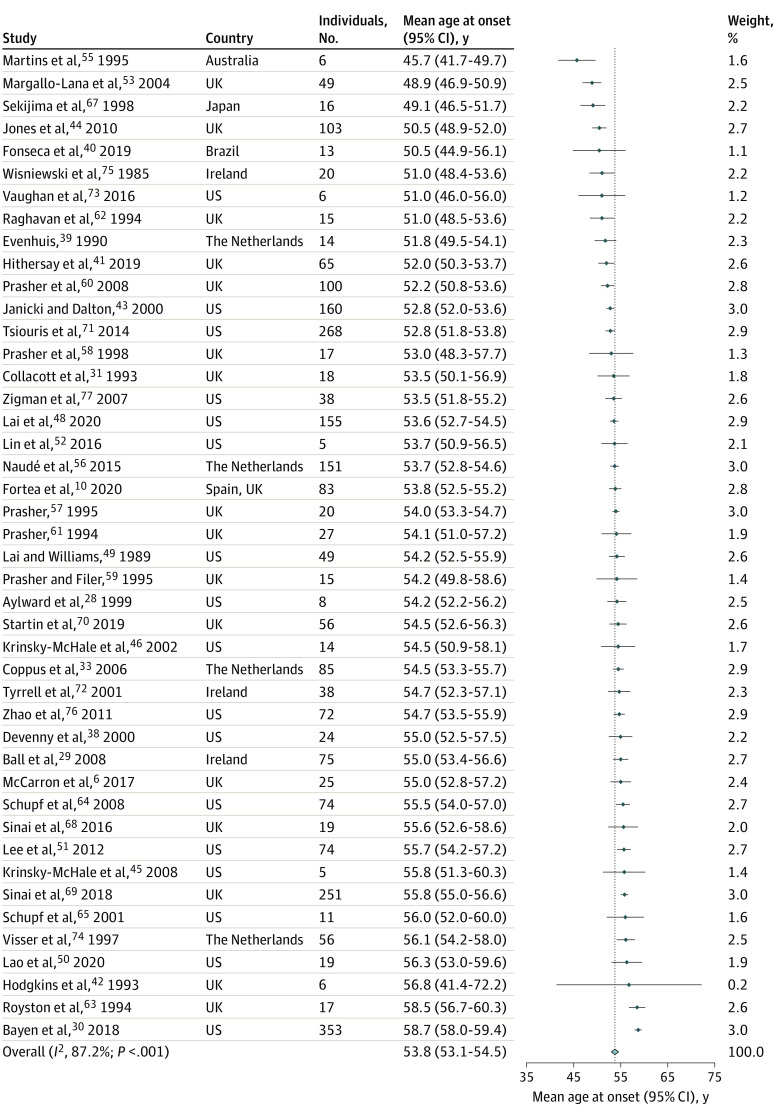
Forest Plot of Mean Age at Onset of Alzheimer Disease Dementia in Adults With Down Syndrome Forest plot of mean age at onset of Alzheimer disease dementia (years) in adults with Down syndrome across 44 eligible studies from the systematic review (N = 2695 individuals). The points represent the mean, the arms indicate the 95% CIs, the light blue diamond indicates the overall pooled estimate (calculated with the DerSimonian-Laird method), and its width indicates the 95% CI. The dotted line indicates the pooled estimate of age at onset (53.8 years). The minimal sample size for a study to be eligible was 5 or more participants. Weights were calculated using random-effects analysis.

**Figure 2.**
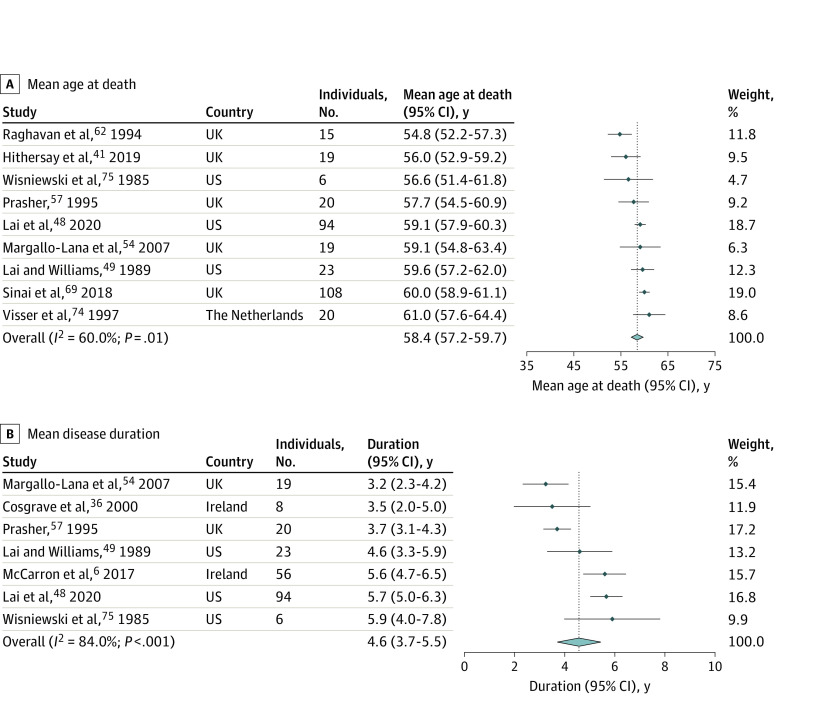
Forest Plot of Mean Age at Death and Mean Duration of Alzheimer Disease Dementia in Adults With Down Syndrome The forest plot shows (A) the mean age at death across 9 eligible studies (N = 324) and (B) the mean disease duration across 7 eligible studies from the systematic review (N = 226 individuals) for Alzheimer disease dementia in adults with Down syndrome. The points indicate the mean, the arms indicate the 95% CI, the light blue diamond represents the overall pooled estimate (calculated with the DerSimonian-Laird method), and its width represents the confidence interval. The dotted line indicates the pooled estimates for age at death (58.4 years) and duration (4.6 years). The minimal sample size for a study to be eligible was 5 or more participants. Weights were calculated using random-effects analysis.

The age at onset of Alzheimer disease dementia in Down syndrome was highly conserved, with a pooled estimate of 53.8 (95% CI, 53.1-54.5) years (n = 2695) (Figure 1). For age at death and disease duration, the pooled estimates were 58.4 (95% CI, 57.2-59.7) (n = 324) and 4.6 (95% CI, 3.7-5.5) years (n = 226), respectively (Figure 2). Although there was considerable statistical heterogeneity in the meta-analysis (*I^2^* = 60%-87%), there was, nevertheless, high consistency in the mean values of the three outcomes across studies. eFigures 3, 4, 5, 6, and 7 in the Supplement show the subanalysis of age at onset by sex, *APOE* genotype, data source, geographic location and sample size. For the 3 outcomes of interest, most studies were ranked at low risk of bias (eTables 5, 6, and 7 in the Supplement) and age at onset did not vary significantly by study quality (eFigure 2 in the Supplement).

#### Variability in the Onset of Alzheimer Disease

**Figure 3.**
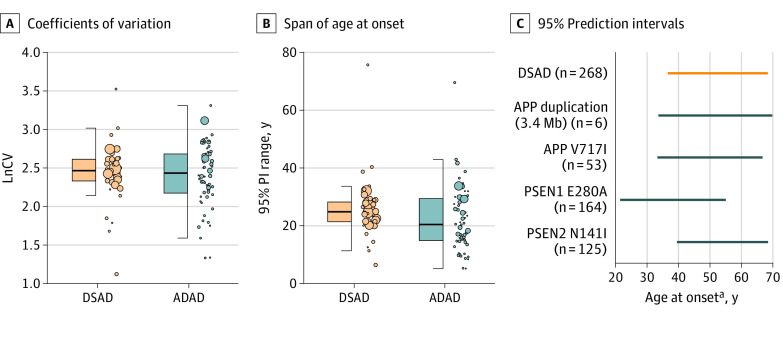
Variability in Age at Onset Panel A shows the natural logarithm of coefficients of variation (LnCV, SD / mean × 100) of the 44 studies on age at onset of Alzheimer disease dementia in adults with Down syndrome (DSAD) included in the systematic review and the LnCV of 60 studies on symptom onset in autosomal dominant Alzheimer disease.^20^ Panel B shows the span of age at onset. The span of age at onset was calculated by computing the 95% prediction interval (PI) range of the same studies included in panel A, by subtracting the lower 95% PI to the upper 95% PI. A larger PI range indicates a wider interval (in years) in which 95% of individuals will develop symptoms. Dots indicate a single study, the size of the dot indicates the sample size, whiskers indicate the 25th and 75th percentiles, and the bold line in the boxes indicate the median. Panel C shows the 95% prediction intervals (PI) for the largest individual cohort studies. For DSAD, the data is from the study of Tsiouris et al.^71^ For ADAD, individual pathogenic variants are indicated in the Figure. ADAD indicates autosomal dominant Alzheimer disease. ^a^Onset refers to the onset of dementia in DSAD and to the onset of progressive cognitive symptoms in ADAD, as determined by clinicians.

Despite differences in sample size, cohort characteristics, and methods, the variability in age at symptom onset between Down syndrome and autosomal dominant Alzheimer disease was comparable both when considering the coefficients of variation and 95% PIs (Figure 3 and eFigure 8 in the Supplement). The interval in which 95% of individuals could develop Alzheimer disease symptoms highly overlapped, spanning the median (IQR) 95% PI range of 24.9 (21.5-28.3) years in Down syndrome and 20.5 (15-29.4) years in autosomal dominant Alzheimer disease (panel B of Figure 3). Panel C of Figure 3 shows the similarities in 95% PI range of age at onset between Down syndrome (largest individual cohort study) and the largest cohorts for each pathogenic variant. Age at onset spanned 36 years in families with the 3.4Mb *APP* duplication, 34 years in the *APP* V717I variant, 32 years in Down syndrome, 33 years in the E280A *PSEN1* variant, and 29 years in the *PSEN2* N141I variant. Of note, sample size influenced the variability observed in autosomal dominant Alzheimer disease but not in Down syndrome (eFigure 9 in the Supplement).

### Mortality Analyses

#### A Hypothetical Distribution of Age at Death Assuming Fully Penetrant Alzheimer Disease

**Figure 4.**
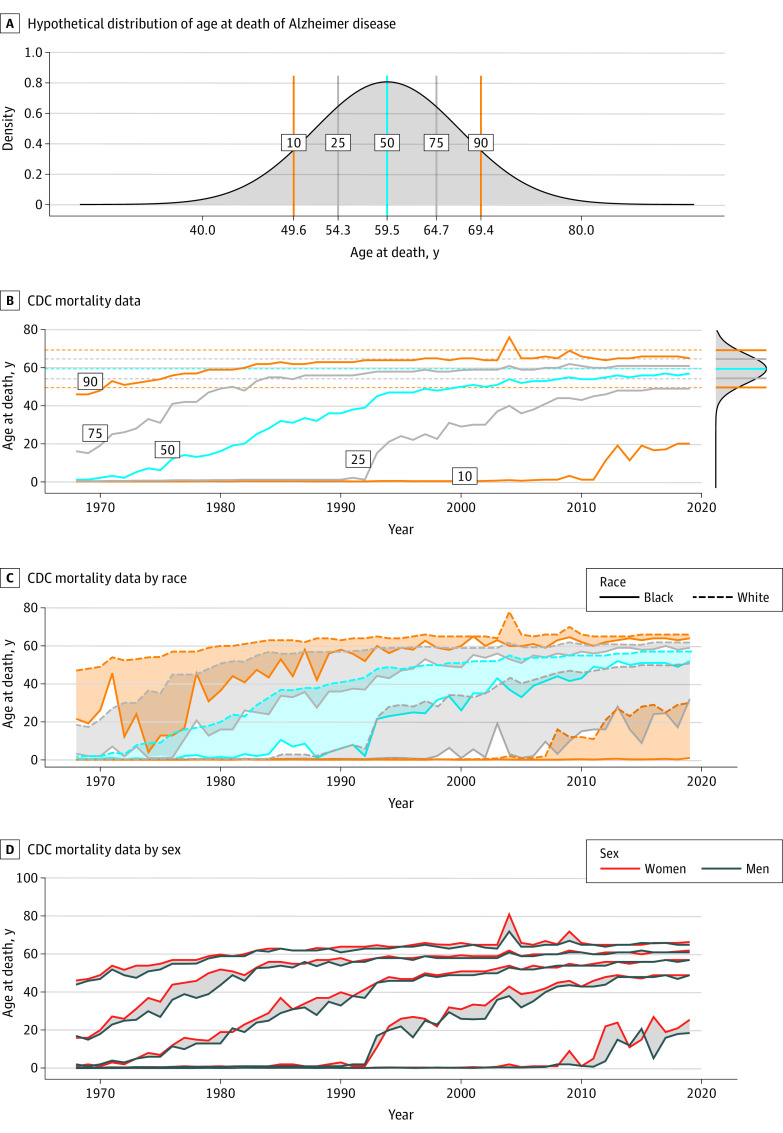
Association of Alzheimer Disease With Life Expectancy in People With Down Syndrome Panel A shows the hypothetical distribution of age at death in people with Down syndrome based on the data on age at onset and disease duration of Alzheimer disease dementia obtained in the systematic review and assuming full Alzheimer disease penetrance. Panel B shows the Centers for Disease Control and Prevention (CDC) mortality data of age at death in individuals with Down syndrome in the US between 1968 and 2019. The graph on the top right side of panel B is an overlay of the predicted percentiles based on the hypothetical distribution shown in panel A. CDC mortality data on age at death stratified by race (Panel C) and sex (Panel D). In panel C, orange lines indicate the 10th and 90th percentiles; gray lines indicate the 25th and 75th percentiles; and blue lines indicate the median. The shaded areas in panels C and D represent differences between each percentile according to race and sex.

Using a combined mean (SD) estimate of age at onset of 54.4 (7.1) years and disease duration of 5.1 (3.1) years calculated from the systematic review, we constructed a hypothetical distribution of age at death in Down syndrome assuming Alzheimer disease had 100% penetrance and was lethal. The estimated 10th, 25th, 50th, 75th, and 90th percentiles in this distribution were 49.6 years, 54.3 years, 59.5 years, 64.7 years, and 69.4 years, respectively (Figure 4). The percentiles were very similar when calculated from the estimate of age at death, corresponding to 50.7 years, 54.6 years, 59 years, 63.4 years, and 67.4 years, respectively (eFigure 10 in the Supplement).

We then contrasted these results with data obtained from US death certificates from 1968 to 2019. We identified 77 347 case records with the respective *ICD* codes for Down syndrome (37 900 females [49%]). We found a remarkable increase in the median age at death of people with Down syndrome in the past 50 years, from 1 year in 1968 to 57 years in 2019 (Figure 4; eFigure 11 in the Supplement). However, we also observed a ceiling effect in the last 2 decades in the higher percentiles, suggesting a limit on life expectancy.

Importantly, these percentiles were in close agreement with our fully penetrant model (Figure 4; eFigure 10 in the Supplement), suggesting that Alzheimer disease is a likely cause for this limit in survival in adults with Down syndrome. We also found racial disparities but no clear sex differences (Figure 4). Age at death was consistently lower for Black individuals, except for the higher percentiles in the 2010s.

#### Contributing Causes of Death in Down Syndrome

**Figure 5.**
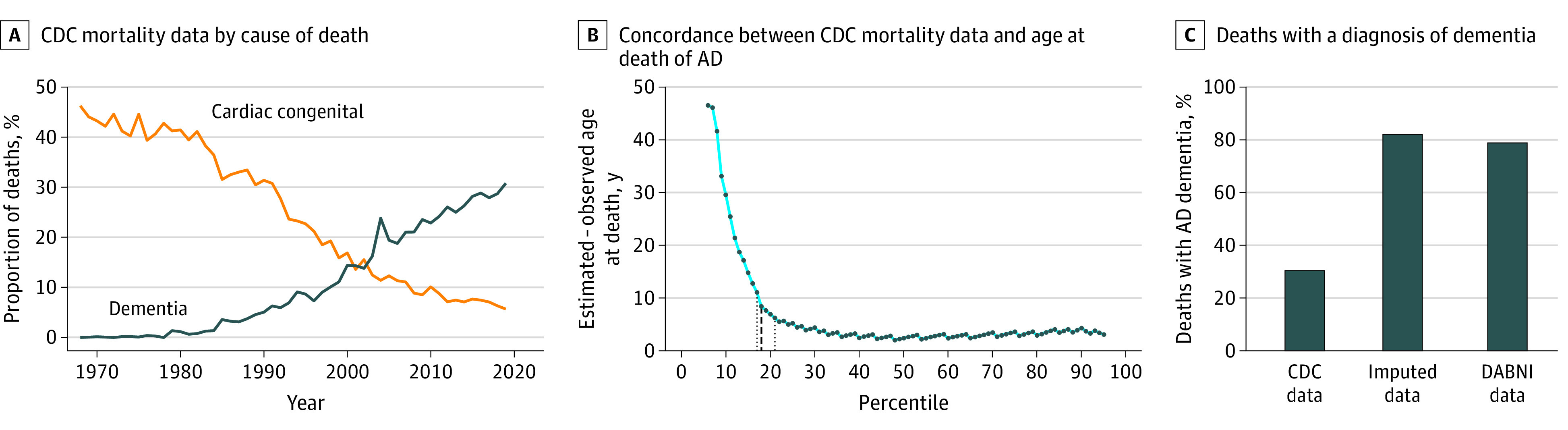
Reporting of Congenital Heart Defects and Dementia in Death Certificates Panel A shows the US Centers for Disease Control and Prevention (CDC) mortality data by cause of death. Results are expressed as the percentage based on the total number of deaths recorded each year. Panel B shows the concordance between the CDC mortality data and the age at death from Alzheimer disease (AD) calculated from the hypothetical death distribution. The black dashed lines indicate the estimate, and the black dotted lines indicate the CI for the inflection point estimated using threshold regressions. Panel C shows the proportion of deaths with a diagnosis of dementia obtained from death certificates at the CDC, from the difference between the estimated and observed age at death (imputed), and from the Down Alzheimer Barcelona Neuroimaging Initiative (DABNI) cohort.

Deaths related to congenital heart disease and dementia were frequent contributing causes of death in Down syndrome in the period 1968 to 2019. Deaths related to congenital heart disease decreased in the last 50 years, while those related to dementia increased up to 30% in 2019 (Figure 5).

To approximate the percentage of deaths potentially linked to Alzheimer disease, we performed 2 analyses. First, we calculated the difference between the hypothetical mortality distribution and the observed ages at death. Whenever the difference was close to 0, we assumed that Alzheimer disease could be the contributor. A larger difference would likely indicate other causes of death. Figure 5 shows the difference between the hypothetical and CDC data for the year 2019 (and eFigure 12 in the Supplement for 1968 to 2019). We can see that the difference between these estimates is larger until approximately the 20th percentile and minimal after. We found a significant inflection point at percentile 18 (95% CI, 17-21), suggesting that up to 80% of deaths in Down syndrome might have Alzheimer disease as a contributing factor.

In addition to the imputed data, we calculated the proportion of deaths with dementia in the DABNI cohort. A total of 889 individuals were included in the final sample, with a mean (SD) age at baseline of 42.4 (11.5) years. Of these, 90 (54 males, 36 females) died during the study period: 71 died with a diagnosis of Alzheimer dementia, 5 with a diagnosis of prodromal Alzheimer disease and 14 were asymptomatic. Therefore, 71 (78.9%) of participants in the DABNI cohort had dementia when they died, in close agreement with the imputed data and in striking contrast with that reported in death certificates. Likewise, there was agreement between the median (IQR) age at death of those with dementia in the DABNI cohort, which was 58.7 (53.5-63.0) years, the estimate of age at death derived from the meta-analysis (58.4 [95% CI, 57.2-59.7] years) and the CDC mortality data (median [IQR] age, 57 [49-61] years). The other percentiles in DABNI (10th, 25th, 75th, 90th) were also congruent with the hypothetical death distribution and the CDC data, and corresponded to 48.3, 53.5, 63.0, and 64.5 years, respectively.

## Discussion

This study suggests that the onset of Alzheimer disease in Down syndrome is as variable as in the autosomal dominant forms. Our findings also suggest that most people with Down syndrome now live up to the limit imposed by Alzheimer disease, suggesting near full penetrance of this disease. Therefore, the lifespan of people with Down syndrome, which is stil approximately 20 years lower than the general population, will not increase further until treatments against Alzheimer disease are available. These findings have considerable implications for public health, policymaking, and counseling to families.

Notably, the common assumption has been that the age at onset of dementia in Down syndrome is highly variable,^13,14,15,16,17,18,19^ in contrast with the autosomal dominant forms, in which age at onset is considered relatively constant within each pedigree.^20^ This view likely stems from the emphasis given to the wide range of disease onset in Down syndrome (ie, 95% PIs) as opposed to the predictability of disease onset ascribed to autosomal dominant forms (ie, 95% CIs). Indeed, given that the diagnosis of symptomatic Alzheimer disease is challenging in Down syndrome, we expected a larger variability in disease onset compared with autosomal dominant Alzheimer disease. However, our analysis revealed that age at onset was consistent across studies, with a similar variability as in the autosomal dominant forms. Importantly, the estimate of age at onset and the variability range was similar to that described in families with *APP* duplications (52.2; 95% CI, 49.9-54.4).^20^ This agrees with the concept that dementia in Down syndrome is primarily driven by the extra copy of the *APP* gene, which is both sufficient and necessary to cause Alzheimer disease pathology and symptoms.^8,12^

While age at onset and its range were consistent across studies, this variability was quite large (25 years). Therefore, it is critical to understand the causes of this variability and to study what modifiable and non-modifiable factors may influence it. For instance, the *APOE* ɛ4 haplotype is associated with a 2-year earlier onset of symptomatic Alzheimer disease in Down syndrome.^78^ Further studies should assess the impact of other medical conditions associated with Down syndrome (eg, hypothyroidism, autoimmune disorders, periodontal disease, sleep apnea, epilepsy, vision and hearing problems) as well as the contribution of sociodemographic factors, such as residential status (living with family vs institutionalization), intellectual disability, and economic status of the caregivers.^68,79^

The main finding of this study is the observation that the real-world mortality data fitted with a distribution of age at death assuming fully penetrant Alzheimer disease. Treating modifiable conditions has dramatically increased life expectancy in Down syndrome in the past decades; however, Alzheimer disease is lethal and has no cure, and therefore imposes a limit on survival for most adults with Down syndrome. In other words, our study may provide an explanation for the gap in life expectancy between Down syndrome and the general population, and the approximately 15-year stagnation of age at death. Our study further expands these results by suggesting that, although Alzheimer disease diminished the differences in life expectancy between Black individuals and White individuals in the higher percentiles, disparities still persist in lower percentiles. As previously suggested, these differences likely reflect avoidable deaths via better access to specialized health services (eg, pediatric cardiology).^80^

The importance of Alzheimer disease as a leading cause of death in Down syndrome is increasingly recognized and is reported in more than 30% of US death certificates in 2019. However, this is likely an underestimate, given that Alzheimer disease is typically underreported in death certificates in the general population. To attempt to quantify this underreporting, we conducted 2 analyses. First, we imputed data between our hypothetical death distribution and the CDC data, which suggested that Alzheimer disease could contribute to death in up to 80% of individuals with Down syndrome. Second, we analyzed mortality data from the DABNI cohort, which revealed that 78.9% of all deaths occurred in those with dementia. Of note, this is consistent with a prior longitudinal cohort study reporting that Alzheimer disease was the proximate cause of death in 70% of adults with Down syndrome.^41^ Altogether, these data reinforce the idea that dementia-related deaths are largely underreported in death certificates of people with Down sydrome.^26,27^

Notwithstanding the importance of other medical comorbidities, which can greatly affect the quality of life of people with Down syndrome, our findings emphasize a strong association between Alzheimer disease and mortality, which could explain the more than 20-year difference in life expectancy compared with the general population. Furthermore, our study underscores that Alzheimer disease has the same variability in age at onset in Down syndrome and autosomal dominant pedigrees. This demonstrates that Down syndrome is also an optimal population to study the natural history of Alzheimer disease.^10,81^ Likewise, the predictability in age at onset (and biomarker changes)^10^ seen in Down syndrome, similar to that seen in autosomal dominant forms, support the use of EYO when comparing clinical and biomarker changes in both genetic forms of dementia and can facilitate the enrolment of persons with presymptomatic disease into clinical trials.^12^ Many efforts are already in place, including the development of large population cohorts gathering clinical and biomarker data on Alzheimer disease,^10,12,13^ and also the launching of trial-ready cohorts across the US and several international sites.

### Limitations

This study had limitations. We performed a systematic search and meta-analysis with strict criteria to ensure rigor and excellent methodological quality; however, diagnostic criteria used for dementia diagnosis varied across studies, and samples sizes were often small. Data were predominantly from European and US data sources, and the number of studies that included data on disease duration and age at death was small. Regarding the CDC mortality data, these were extracted from death certificates, which have inherent limitations, such as incomplete reporting, particularly of co-occurring conditions contributing to death. We overcame this limitation by also analyzing mortality data from a prospective clinical cohort (ie, DABNI) designed to study the natural history of Alzheimer disease in Down syndrome. As most participants in the DABNI cohort are of Caucasian ethnicity, we could not perform segregated analyses to match those presented with the CDC data.

## Conclusions

These findings suggests that Alzheimer disease should be recognized as a critical medical priority in people with Down syndrome. The mortality data suggest that Down syndrome is compatible with fully penetrant Alzheimer disease. Therefore, increasing life expectancy and closing the gap with the general population will require effective prevention and management for Alzheimer disease.

## Notes

Supplement. **eAppendix.** Methods and Results **eTable 1. **Pubmed Search Strategy and Hits Retrieved **eTable 2. **Quality and Rigor Assessment of Included Studies **eTable 3.** Extraction of Death Certificate Data of Down Syndrome From CDC Records **eTable 4.** Summary Characteristics of Eligible Studies **eTable 5.** Extracted Data From 44 Studies on Age at Onset of Alzheimer Disease **eTable 6.** Extracted Data From 9 Studies on Age at Death of Alzheimer Disease **eTable 7.** Extracted Data From 7 Studies on Duration of Alzheimer Disease **eFigure 1.** PRISMA Flow Diagram **eFigure 2.** Association of Risk of Bias and Age at Onset Estimate **eFigure 3.** Association of Biological Sex and Age at Onset Estimate **eFigure 4.** Association of *APOE* Genotype and Age at Onset Estimate **eFigure 5.** Association of Data Source and Age at Onset Estimate **eFigure 6.** Association of Geographic Location and Age at Onset Estimate **eFigure 7.** Association of Sample Size With Age at Onset Estimate **eFigure 8.** Variability in Age at Onset **eFigure 9.** Effect of Sample Size on Variability Estimates **eFigure 10.** Concordance Between Estimated and Real Distribution of Age at Death **eFigure 11.** Distribution of Age at Death in Persons With Down Syndrome in the USA **eFigure 12.** Difference Between the Estimated and Observed Age at Death of People With Down Syndrome in the USA Between 1968-2018
eReferences.
