# Supplementary material for: Association of Alzheimer Disease With Life Expectancy in People With Down Syndrome
Source: JAMA Netw Open. 2022 May 23;5(5):e2212910. doi: 10.1001/jamanetworkopen.2022.12910 (PMC9127560; doi:10.1001/jamanetworkopen.2022.12910)
Supplement: Supplement. — eAppendix. Methods and Results eTable 1. Pubmed Search Strategy and Hits Retrieved eTable 2. Quality and Rigor Assessment of Included Studies eTable 3. Extraction of Death Certificate Data of Down Syndrome From CDC Records eTable 4. Summary Characteristics of Eligible Studies eTable 5. Extracted Data From 44 Studies on Age at Onset of Alzheimer Disease eTable 6. Extracted Data From 9 Studies on Age at Death of Alzheimer Disease eTable 7. Extracted Data From 7 Studies on Duration of Alzheimer Disease eFigure 1. PRISMA Flow Diagram eFigure 2. Association of Risk of Bias and Age at Onset Estimate eFigure 3. Association of Biological Sex and Age at Onset Estimate eFigure 4. Association of APOE Genotype and Age at Onset Estimate eFigure 5. Association of Data Source and Age at Onset Estimate eFigure 6. Association of Geographic Location and Age at Onset Estimate eFigure 7. Association of Sample Size With Age at Onset Estimate eFigure 8. Variability in Age at Onset eFigure 9. Effect of Sample Size on Variability Estimates eFigure 10. Concordance Between Estimated and Real Distribution of Age at Death eFigure 11. Distribution of Age at Death in Persons With Down Syndrome in the USA eFigure 12. Difference Between the Estimated and Observed Age at Death of People With Down Syndrome in the USA Between 1968-2018 eReferences. [file jamanetwopen-e2212910-s001.pdf]

Supplemental Online Content

Iulita MF, Garzón Chavez D, Klitgaard Christensen M, et al. Association of Alzheimer disease with life expectancy in people with Down syndrome. *JAMA Netw Open*. 2022;5(5):e2212910. doi:10.1001/jamanetworkopen.2022.12910

eAppendix. Methods and Results
eTable 1. Pubmed Search Strategy and Hits Retrieved
eTable 2. Quality and Rigor Assessment of Included Studies
eTable 3. Extraction of Death Certificate Data of Down Syndrome From CDC Records
eTable 4. Summary Characteristics of Eligible Studies
eTable 5. Extracted Data From 44 Studies on Age at Onset of Alzheimer Disease
eTable 6. Extracted Data From 9 Studies on Age at Death of Alzheimer Disease
eTable 7. Extracted Data From 7 Studies on Duration of Alzheimer Disease
eFigure 1. PRISMA Flow Diagram
eFigure 2. Association of Risk of Bias and Age at Onset Estimate
eFigure 3. Association of Biological Sex and Age at Onset Estimate
eFigure 4. Association of *APOE* Genotype and Age at Onset Estimate
eFigure 5. Association of Data Source and Age at Onset Estimate
eFigure 6. Association of Geographic Location and Age at Onset Estimate
eFigure 7. Association of Sample Size With Age at Onset Estimate
eFigure 8. Variability in Age at Onset
eFigure 9. Effect of Sample Size on Variability Estimates
eFigure 10. Concordance Between Estimated and Real Distribution of Age at Death
eFigure 11. Distribution of Age at Death in Persons With Down Syndrome in the USA
eFigure 12. Difference Between the Estimated and Observed Age at Death of People With Down Syndrome in the USA Between 1968-2018
eReferences.

This supplemental material has been provided by the authors to give readers additional information about their work.

© 2022 Iulita MF et al. *JAMA Network Open.*

eAppendix. Methods and Results

Methods

Systematic review and meta-analysis

We conducted a systematic search to examine the age at onset, age at death, and duration of Alzheimer disease among persons with Down syndrome. The terms "age at onset" and "age at diagnosis" were considered interchangeable. The following databases were used: PubMed/Medline, Embase, Web of Science, and CINAHL. OpenGray was used for gray literature. A medical librarian was consulted for the development of the search strategy (eTable 1). The first search was done on August 10, 2020, and a final search was completed on February 7, 2021.

Study selection and data extraction

Only peer-reviewed, original research studies written in English were eligible. No time restrictions were applied. We excluded pre-prints, reviews, abstracts, case reports, systematic reviews, or articles with a sample size <5. We excluded studies not available for full-text review after searching in three different international libraries. Regarding the full-text review process, a database compiling all decisions was created and the reason(s) for exclusion of a study were recorded. Data extraction of eligible studies was performed independently by each reviewer (MFI and DGC) and then compared for consensus. Besides the primary outcome data, we also included variables on the data source (population cohort or clinical/epidemiological records), study type (cross-sectional, longitudinal), geographic location, as well as details on the patient population including sample size, sex distribution, and availability of karyotype and/or *APOE* genotype.

Quality and risk of bias

We employed a quality assessment tool (eTable 2), adapted from the scale of McGrath and colleagues[1] to assess the methodological quality of eligible studies. Studies were assigned quality points (0, 1, 2, 3) for certain methodological characteristics and/or data descriptors (data source, quality of method for Alzheimer disease diagnosis, use of diagnostic criteria, confirmation of Down syndrome by genetic screening, sample size, number of outcomes available). The maximum was 12 points. Two researchers (MFI, DGC) performed this assessment independently.

Risk of bias of included studies was assessed using the quality scale to classify studies into low, moderate, and high risk of bias. Cohort studies, with face-to-face diagnostic interviews, and large sample sizes were considered at low risk of bias (quality score 10-12); studies with data from clinical records with a diagnostic assignment based on hospital notes or case note reviews were considered at moderate risk (score 8-9) and studies with data and diagnoses from epidemiological registries were considered at high risk of bias (score 6-7).

Data synthesis and analysis

When the outcome of interest was found in the same cohort across different studies, the largest cohort was considered for data synthesis and analysis. When a study only provided data on individual subgroups (e.g., men and women), a weighted mean and standard deviation for the combined sample were calculated, based on the formula for combining groups stated in the Cochrane Handbook for Systematic Reviews of Interventions. Interventions.[2]

Analyses of subgroups were conducted to examine the influence of study-level characteristics on the overall estimate of age at Alzheimer disease onset (e.g., data from population-based or convenience cohorts versus clinical records/registries, geographic location, sex, *APOE* genotype). These analyses were not performed for age at death and disease duration due to the limited number of studies (9 and 7 studies, respectively). Except for data concerning our population-based study[3], which was accessible

© 2022 Iulita MF et al. *JAMA Network Open.*

to us, authors were not contacted to seek additional information on sex or *APOE* genotype if they were not available in the published study.

Results

Systematic review and meta-analysis

Description of included studies

A total of 44 studies had data on age at onset, with 38 (86%) containing data from population-based or convenience cohorts, and 6 (14%) from epidemiological registries or clinical records. Alzheimer disease diagnosis was based on specific clinical criteria (68%) or expert clinical judgement (32%). A total of 2595 individuals were included (median of 26 individuals per study; range 5-353). 53% were women and 12 studies did not have this information available. Additional study characteristics including confirmation of trisomy 21, study region, and design are described in eTable 4).

We found data on age at death and disease duration of Alzheimer disease in 9 and 7 studies, respectively, mainly from population-based or convenience cohorts. We included a total of 324 and 226 individuals, respectively (median of 20 individuals per study; range 6-108). 53% were women, and 3 studies did not have this information available. Further study details are available in eTable 4.

All the included studies had good methodologically quality and rigor, and risk of bias was assessed in all studies individually (eTables 5-7). For the three outcomes of interest (onset, death, duration), most studies (52%, 67%, 71%, respectively) were ranked at low risk of bias. Of note age at onset did not vary significantly between studies ranked at high, low or moderate risk of bias (eFigure 2).

We assessed the influence of biological sex, *APOE* genotype and data source on age at onset. Females and males had similar ages at onset (53.9 vs. 54.3 years) (eFigure 3). The estimate for age at onset was lower in *APOE* ε4 carriers than in non-carriers (51.4 vs. 53.1 years) (eFigure 4) and in studies of population cohorts vs. registry data or clinical records (53.5 vs. 55.5 years) (eFigure 5). eFigure 6 also shows a sub-analysis by geographic location.

To formally investigate if the sample size the studies affected the estimate of age at onset, we computed the meta-analysis estimates iteratively using subsets of studies based on their sample size. As can be seen in the eFigure 7, the estimates of age at onset were very comparable, regardless of the sample size of the study.

Mortality analyses

US mortality data

We selected all cases with ICD codes for Down syndrome listed as the underlying cause of death and in the record axis. We excluded records that contained a code for abortion or pregnancy termination and 0 years (eTable 3). We retrieved demographic information (sex, race) and up to 20 health conditions listed on the death certificates. To evaluate potential race differences, we compared data between individuals based on the race identified on the record (black or white).

Diagnostic evaluation in the DABNI cohort

DABNI is a prospective longitudinal cohort to screen for Alzheimer disease in Down syndrome. All adults with Down syndrome living in Catalonia are invited to participate and the recruitment is part of a public health plan developed to screen and early diagnose Alzheimer disease.

Prior to data collection, all participants or their legal representatives were required to give written informed consent. The diagnostic classification of study participants was performed independently, by

© 2022 Iulita MF et al. *JAMA Network Open.*

the neuropsychologists and neurologists who assessed them, and it was debated during a consensus meeting masked to biomarker data. Functional status to differentiate prodromal Alzheimer disease and Alzheimer disease dementia was assessed on the basis of anamnesis, the Dementia Questionnaire for Persons with Mental retardation, and the CAMDEX-DS (CAMDEX-DS: The Cambridge Examination for Mental Disorders of Older People with Down's Syndrome and Others with Intellectual Disabilities) to differentiate decline due to cognitive impairment from pre-existing intellectual disability, placing a particular emphasis on establishing change from the individual's best level of functioning.

A diagnosis of asymptomatic was given when there was no clinical or neuropsychological suspicion of Alzheimer disease (i.e., absence of cognitive impairment beyond the intellectual and developmental disabilities, or functional decline compared to the previous functioning).

A diagnosis of prodromal Alzheimer disease was given when there was suspicion of Alzheimer disease, but symptoms did not fulfill criteria for dementia (i.e., evidence of cognitive impairment without any functional changes).

A diagnosis of Alzheimer disease dementia required evidence of cognitive impairment beyond the intellectual and developmental disabilities and that interfered with everyday activities (i.e., presence of a functional decline compared to previous functioning).

The diagnostic procedures used in our clinic follow the recommendations of the "Working Group for the Establishment of the Criteria for the Diagnosis of Dementia in Individuals with Developmental Disability".

The mortality data was obtained based on a survey with the family and review of death certificate records and medical records when available. We used the diagnosis of the last follow-up to identify dementia status.

© 2022 Iulita MF et al. *JAMA Network Open.*

eTable 1. Pubmed Search Strategy and Hits Retrieved

Search	Query	Hits
1	"Down Syndrome"[Mesh]	23665
2	((("Down Syndrome"[Mesh]) OR (Down* syndrome)) OR (syndrome, Down*)) OR (trisomy 21)	43138
3	"Alzheimer Disease"[Mesh]	85762
4	(((((((((("Alzheimer Disease"[Mesh]) OR (Alzheimer* disease)) OR (dementia)) OR (dementia, Alzheimer* type)) OR (Alzheimer* type dementia)) OR (Alzheimer*-Type dementia)) OR (Alzheimer* dementia)) OR (presenile dementia)) OR (early onset Alzheimer* disease)) OR (Alzheimer* disease, early onset)) OR (presenile Alzheimer* dementia)	203513
5	(((("Down Syndrome"[Mesh]) OR (Down* syndrome)) OR (syndrome, Down*)) OR (trisomy 21) AND (humans[Filter])) AND ((((((((((("Alzheimer Disease"[Mesh]) OR (Alzheimer* disease)) OR (dementia)) OR (dementia, Alzheimer* type)) OR (Alzheimer* type dementia)) OR (Alzheimer*-type dementia)) OR (Alzheimer* dementia)) OR (presenile dementia)) OR (early onset Alzheimer* disease)) OR (Alzheimer* disease, early onset)) OR (presenile Alzheimer* dementia) AND (humans[Filter]))	2729

Restrictions applied: human studies. Initial search: August 10, 2021. A final search was carried out on February 7, 2021, retrieving a total of 2742 records to screen. The Pubmed search string was adapted to fit the syntax criteria of each of the other search databases.

© 2022 Iulita MF et al. *JAMA Network Open.*

eTable 2. Quality and Rigor Assessment of Included Studies

	**Points**
**Source of patient data**	
Clinical cohort (population-based; convenience)	3
Hospital or clinical records	2
Epidemiological registries	1
Not specified	0
**Diagnosis**	
Diagnostic system reported (e.g., DSM, ICD, NIA-AA, NINDS-ADRDA, local guidelines)	2
Own system/symptoms adequately and systematically described	1
Insufficient details on diagnostic criteria (exclusion criteria)	0
**Method of diagnostic assignment**	
Diagnostic interview (face to face)	3
Case note review	2
Diagnosis recorded in hospital notes or registries	1
Not specified	0
**Patient selection**	
Karyotype confirmation of chromosome 21 trisomy	1
No karyotype confirmation/not specified	0
**Sample size**	
Sample size > 50	1
Sample size <50	0
**Additional 'merits'**	
Data available on both outcomes (age of onset/diagnosis and age at death/duration)	2
Data available on only one outcome	1
**Total**	12

For risk of bias assessment, quality scores 10-12 were considered low risk, scores 8-9 were considered moderate risk and scores 6-7 were considered high risk. Age at onset/diagnosis and duration refer to the onset and duration of Alzheimer´s disease dementia. Age at death refers to those who died with a diagnosis of Alzheimer disease dementia. DSM = Diagnostic and Statistical Manual of Mental Disorders; ICD = International Classification of Diseases; NIA-AA = National Institute on Aging and Alzheimer's Association; NINDS-ADRDA = National Institute of Neurological and Communicative Disorders and Stroke and the Alzheimer's Disease and Related Disorders Association.

© 2022 Iulita MF et al. *JAMA Network Open.*

eTable 3. Extraction of Death Certificate Data of Down Syndrome From CDC Records

A) Flowchart of included and excluded records

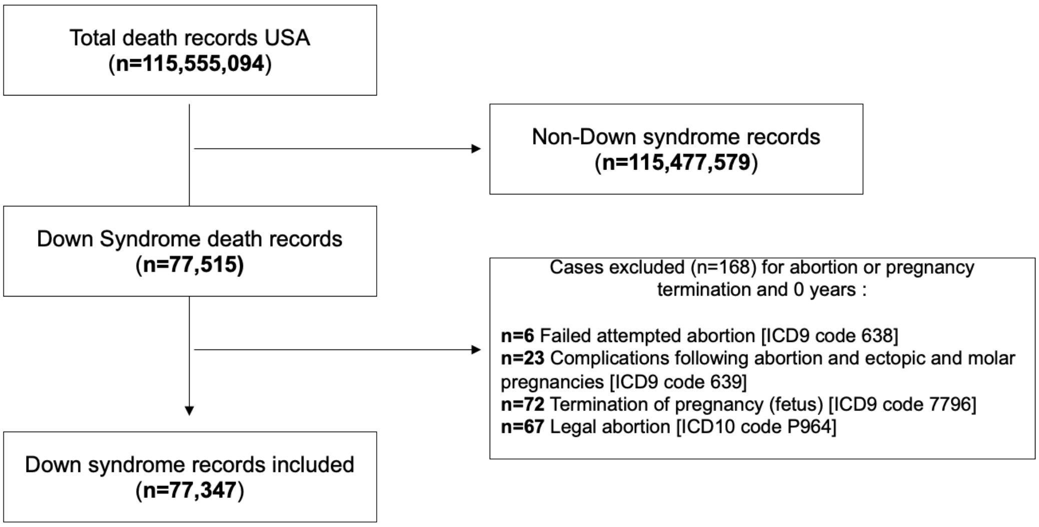

B) Deaths associated with specific health conditions in Down syndrome

Health condition	ICD codes	ICD version	Total records
Abortion	640-645	ICD8	0
Missed abortion	632	ICD9	0
Legal abortion	P964	IC10	67
Termination of pregnancy	773; 779.6	ICD8, ICD9	72
Pregnancy with abortive outcome	634-639	ICD9	29
	O00-O08	ICD10	0
Congenital anomalies of heart	745.0-747.9	ICD8, ICD9	13259
	Q20.0-Q28.9	ICD10	4554
Dementia/Alzheimer disease	331.0-331.9	ICD8, ICD9	840
	290.0-290.9	ICD8, ICD9	320
	294.0-294.9	ICD8, ICD9	117
	G30	ICD10	4654
	F03	ICD10	5078

ICD = International classification of diseases

eTable 4. Summary Characteristics of Eligible Studies

Study characteristics, N (%)	Onset	Death	Duration
Total eligible studies	44	9	7
**Data source**			
Cohort	38 (86)	6 (67)	6 (86)
Records/registries	6 (14)	3 (33)	1 (14)
**Study design**			
Cross-sectional	23 (52)	2 (22)	1 (14)
Longitudinal	21 (48)	7 (78)	6 (86)
**Diagnosis**			
Defined criteria	30 (68)	4 (44)	4 (57)
Clinical judgement	14 (32)	5 (56)	3 (43)
**Karyotype T21**			
Yes	28 (64)	6 (67)	3 (43)
No	16 (36)	3 (33)	4 (57)
**Region**			
Europe	24 (55)	6 (67)	4 (57)
North America	17 (39)	3 (33)	3 (43)
South America	1 (2)	0	0
Asia	1 (2)	0	0
Oceania	1 (1)	0	0
**Quality points**			
10-12	23 (52)	6 (67)	5 (71)
8-9	13 (30)	1 (11)	1 (14)
6-7	8 (18)	2 (22)	1 (14)

© 2022 Iulita MF et al. *JAMA Network Open.*

eTable 5. Extracted Data From 44 Studies on Age at Onset of Alzheimer Disease

study	country	data_source	%_female	mean_onset	SD	N	quality_rate	ROB
Martins RN, et al. 1995	Australia	cohort	50	45.7	5	6	6	High
Margallo-Lana M, et al. 2004	UK	cohort	NA	48.9	7.2	49	10	Low
Sekijima Y, et al. 1998	Japan	cohort	56	49.1	5.4	16	9	Moderate
Jones EL, et al. 2011	UK	cohort	NA	50·5	7.9	103	10	Low
Fonseca LM, et al. 2019	Brazil	cohort	23	50.5	10.3	13	9	Moderate
Wisniewski KE, et al. 1985	Ireland	cohort	75	51.0	6.0	20	7	High
Vaughan RM, et al. 2016	USA	cohort	17	51.0	6.2	6	10	Low
Raghavan R, et al. 1994	UK	clinical records	53	51.0	5.1	15	7	High
Evenhuis HM, et al 1990	Netherlands	cohort	57	51.8	4.4	14	11	Low
Hithersay R, et al. 2019	UK	cohort	55	52.0	7.1	65	12	Low
Prasher VP et al. 2008	UK	cohort	43	52.2	7.1	100	11	Low
Janicki MP, et al. 2000	USA	cohort	NA	52.8	5.2	160	11	Low
Tsiouris JA, et al. 2014	USA	cohort	55	52.8	8.2	268	8	Moderate
Prasher VP, et al. 1998	UK	cohort	71	53.0	9.9	17	9	Moderate
Colacott RA, et al. 1993	UK	clinical records	NA	53.5	7.4	18	6	High
Zigman WB, et al. 2007	USA	cohort	71	53.5	5.5	38	10	Low
Lai F, et al. 2020	USA	cohort	37	53.6	5.8	155	10	Low
Lin AL, et al. 2016	USA	cohort	100	53.7	3.2	5	9	Moderate
Naude PJ, et al. 2015	Netherlands	cohort	39	53.7	5.6	151	11	Low
Fortea J, et al. 2020	Spain, UK	cohort	47	53.8	6.2	83	11	Low
Prasher VP, et al. 1995a	UK	clinical records	60	54.0	1.7	20	7	High
Prasher VP, et al. 1994	UK	cohort	59	54.1	8.3	27	10	Low
Lai F, et al. 1989	USA	cohort	45	54.2	6.1	49	10	Low
Prasher VP, et al. 1995b	UK	cohort	80	54.2	8.6	15	10	Low
Aylward EH, et al. 1999	USA	cohort	50	54.2	2.9	8	9	Moderate
Startin CM, et al. 2019	UK	cohort	NA	54.5	7.0	56	11	Low
Krinsky-McHale SJ, et al. 2002	USA	cohort	71	54.5	6.9	14	9	Moderate
Coppus A, et al. 2006	Netherlands	cohort	41	54.5	5.5	85	11	Low
Tyrell J, et al. 2001	Ireland	cohort	NA	54.7	7.5	38	10	Low
Zhao Q, et al. 2011	USA	cohort	100	54.7	5.1	72	11	Low
Devenny DA, et al. 2000	USA	cohort	NA	55.0	6.3	24	9	Moderate
Ball SL, et al. 2008	Ireland	cohort	100	55.0	7.1	75	11	Low
McCarron M, et al. 2017	UK	cohort	NA	55.0	5.6	25	9	Moderate
Schupf N, et al. 2008	USA	cohort	100	55.5	6.5	74	10	Low
Sinai A, et al. 2016	UK	cohort	37	55.6	6.8	19	9	Moderate
Lee JH, et al. 2012	USA	cohort	100	55.7	6.4	74	10	Low
Krinsky-McHale SJ, et al. 2008	USA	cohort	NA	55.8	5.1	5	10	Low
Sinai A, et al. 2018	UK	clinical records	45	55.8	6.3	251	8	Moderate
Schupf N, et al. 2001	USA	cohort	NA	56.0	6.7	11	9	Moderate
Visser FE, et al. 1997	Netherlands	cohort	NA	56.1	7.4	56	11	Low
Lao PJ, et al. 2020	USA	cohort	47	56.3	7.2	19	8	Moderate
Hodgkins PS, et al. 1993	UK	cohort	33	56.8	19.3	6	7	High
Royston MC, et al. 1994	UK	clinical records	NA	58.5	3.7	17	7	High
Bayen E, et al. 2018	USA	clinical records	49	58.7	7.1	353	6	High

© 2022 Iulita MF et al. *JAMA Network Open.*

Mean_onset = mean age at Alzheimer disease onset or diagnosis (years) in adults with Down syndrome; N = sample size; NA = not available; SD = standard deviation; ROB = risk of bias. The term cohort represents population-based or convenience cohorts. The term clinical records refers to data extracted from epidemiological registers, medical files and/or hospital records. The maximum number of points in the quality scale is 12.

© 2022 Iulita MF et al. *JAMA Network Open.*

eTable 6. Extracted Data From 9 Studies on Age at Death of Alzheimer Disease

study	country	data_source	% female	mean_death	SD	N	quality_rate	ROB
Raghavan R, et al. 1994	UK	clinical records	53	54.8	5.0	15	7	High
Hithersay R, et al. 2019	UK	cohort	47	56.1	7.0	19	12	Low
Wisniewski KE, et al. 1985	USA	cohort	17	56.6	6.5	6	10	Low
Prasher VP, et al. 1995a	UK	clinical records	60	57.7	7.4	20	7	High
Lai F, et al. 2020	USA	cohort	35	59.1	5.8	94	10	Low
Margallo-Lana M, et al. 2007	UK	cohort	NA	59.1	9.6	19	11	Low
Lai F, et al. 1989	USA	cohort	39	59.6	6.0	23	10	Low
Sinai A, et al. 2018	UK	clinical records	45	60.0	6.0	108	8	Moderate
Visser FE, et al. 1997	Netherlands	cohort	NA	61.0	7.8	20	11	Low

Mean_death = mean age at death (years) in those with a diagnosis of Alzheimer disease dementia among adults with Down syndrome; N = sample size; NA = not available; SD = standard deviation; ROB = risk of bias. The term cohort represents population-based or convenience cohorts. The term clinical records refers to data extracted from epidemiological registers, medical files and/or hospital records. The maximum number of points in the quality scale is 12.

© 2022 Iulita MF et al. *JAMA Network Open.*

eTable 7. Extracted Data From 7 Studies on Duration of Alzheimer Disease

study	country	data_source	%_female	mean_duration	SD	N	quality_rate	ROB
Margallo-Lana M, et al. 2007	UK	cohort	NA	3.2	2.0	19	11	Low
Cosgrave MP, et al. 2000	Ireland	cohort	100	3.5	2.2	8	9	Moderate
Prasher VP, et al. 1995a	UK	clinical records	60	3.7	1.3	20	7	High
Lai F, et al. 1989	USA	cohort	39	4.6	3.2	23	10	Low
McCarron M, et al. 2017	Ireland	cohort	100	5.6	3.3	56	11	Low
Lai F, et al. 2020	USA	cohort	35	5.7	3.2	94	10	Low
Wisniewski KE, et al. 1985	USA	cohort	17	5.9	2.4	6	10	Low

Mean_duration = mean duration (years) from Alzheimer disease onset or diagnosis to death in adults with Down syndrome; N = sample size; NA = not available; SD = standard deviation; ROB = risk of bias. The term cohort represents population-based or convenience cohorts. The term clinical records refers to epidemiological registers, medical files and/or hospital records. The maximum number of points in the quality scale is 12.

© 2022 Iulita MF et al. *JAMA Network Open.*

eFigure 1. PRISMA Flow Diagram

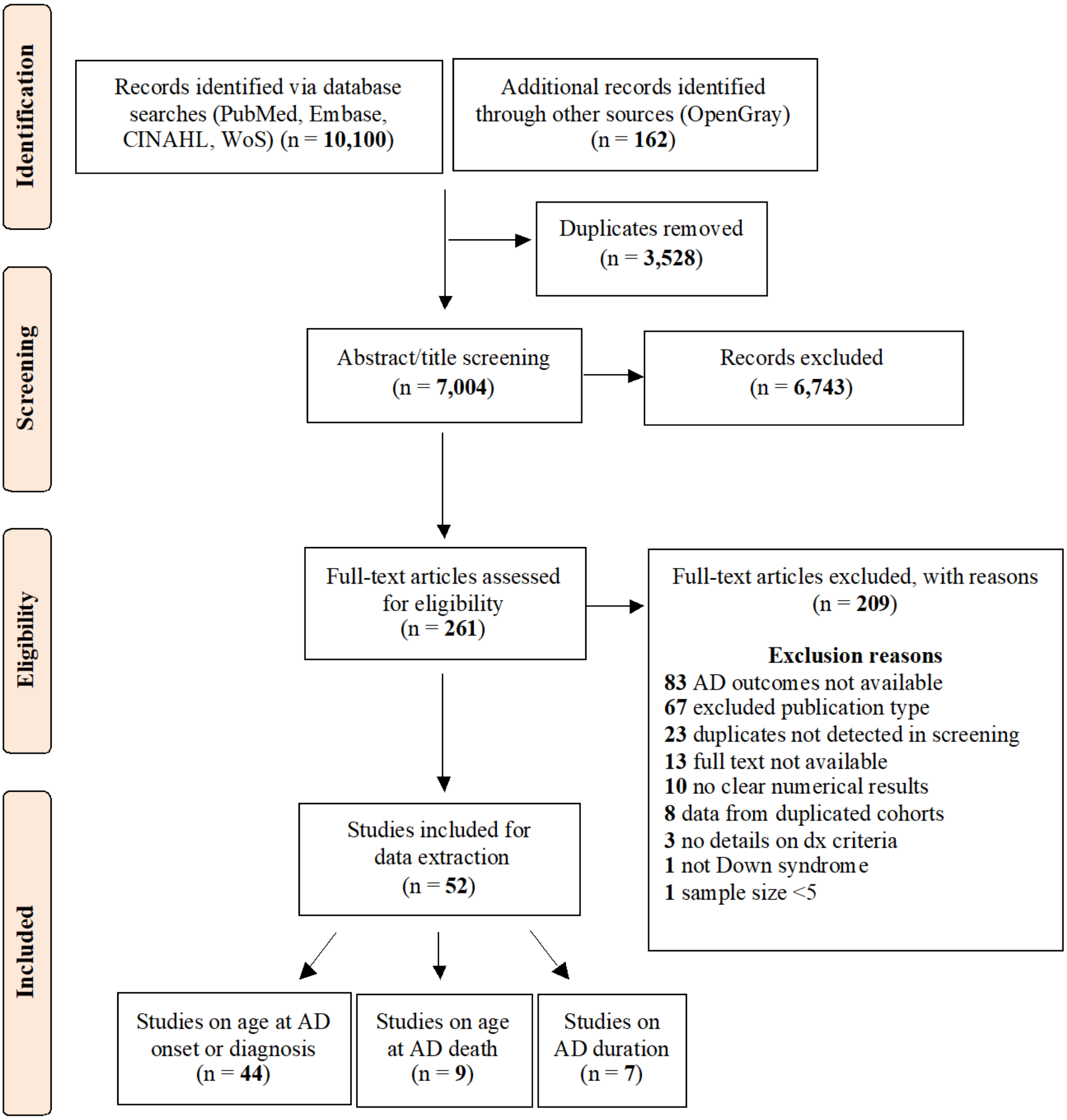

eFigure 1. PRISMA flow diagram adapted from Page et al. Systematic Reviews (2021) 10:89. AD = Alzheimer disease. Note that some eligible studies contained information on more than one outcome of interest (onset, duration, death).

© 2022 Iulita MF et al. *JAMA Network Open.*

eFigure 2. Association of Risk of Bias and Age at Onset Estimate

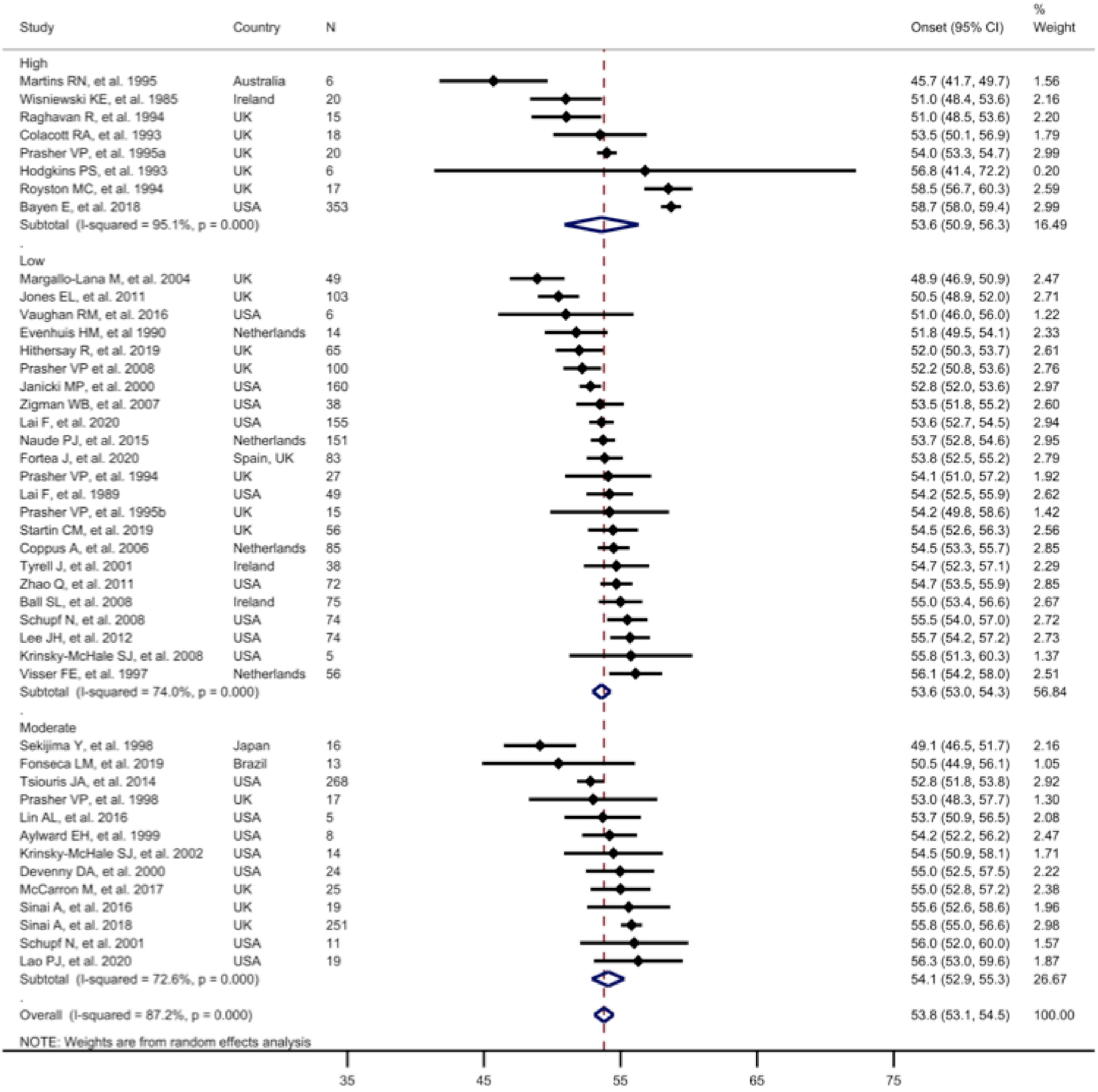

eFigure 2. Forest plot of mean age at onset or diagnosis (years) of Alzheimer disease dementia in adults with Down syndrome according to risk of bias assessment. The black diamond represents the mean with the arms reflecting the 95% confidence intervals (CIs). The unfilled blue diamond represents the overall pooled estimate, and its width represents the confidence interval.

© 2022 Iulita MF et al. *JAMA Network Open.*

eFigure 3. Association of Biological Sex and Age at Onset Estimate

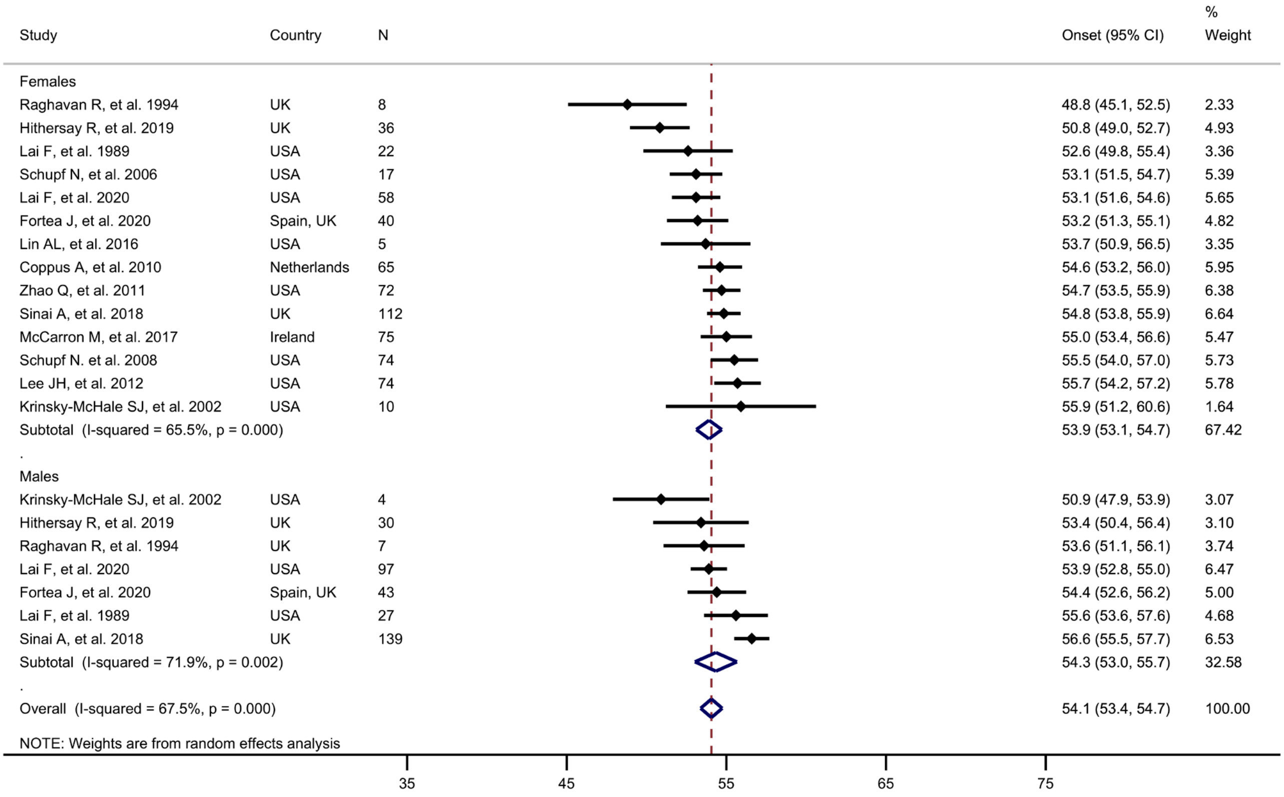

eFigure 3. Forest plot of mean age at onset or diagnosis (years) of Alzheimer disease dementia in adults with Down syndrome according to biological sex. The black diamond represents the mean with the arms reflecting the 95% confidence intervals (CIs). The unfilled blue diamond represents the overall pooled estimate, and its width represents the confidence interval.

© 2022 Iulita MF et al. *JAMA Network Open.*

eFigure 4. Association of *APOE* Genotype and Age at Onset Estimate

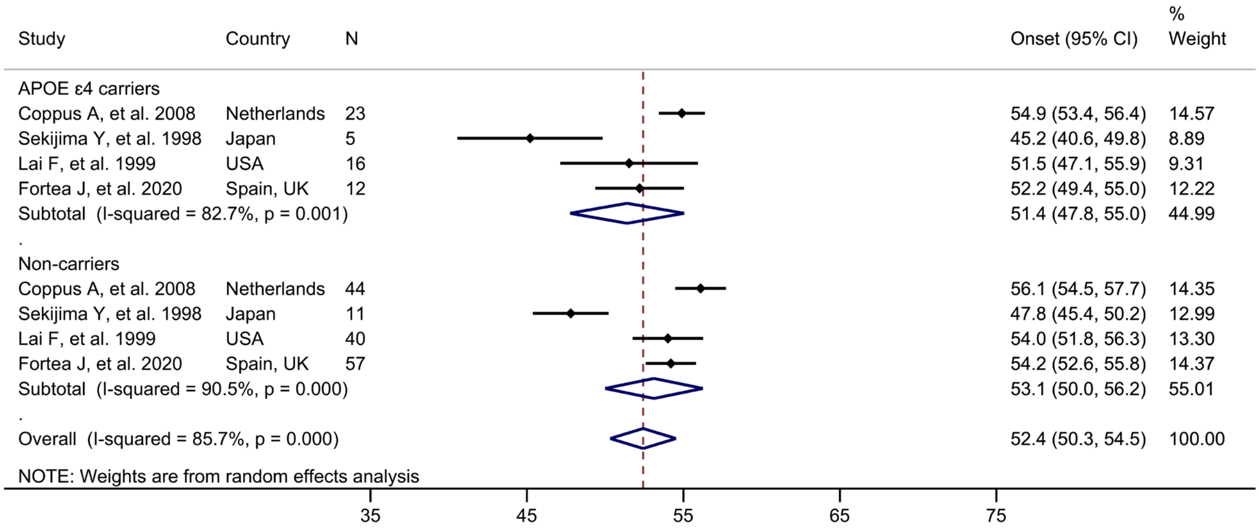

eFigure 4. Forest plot of mean age at onset or diagnosis (years) of Alzheimer disease dementia in adults with Down syndrome according to *APOE* genotype. The black diamond represents the mean with the arms reflecting the 95% confidence intervals (CIs). The unfilled blue diamond represents the overall pooled estimate, and its width represents the confidence interval.

© 2022 Iulita MF et al. *JAMA Network Open.*

eFigure 5. Association of Data Source and Age at Onset Estimate

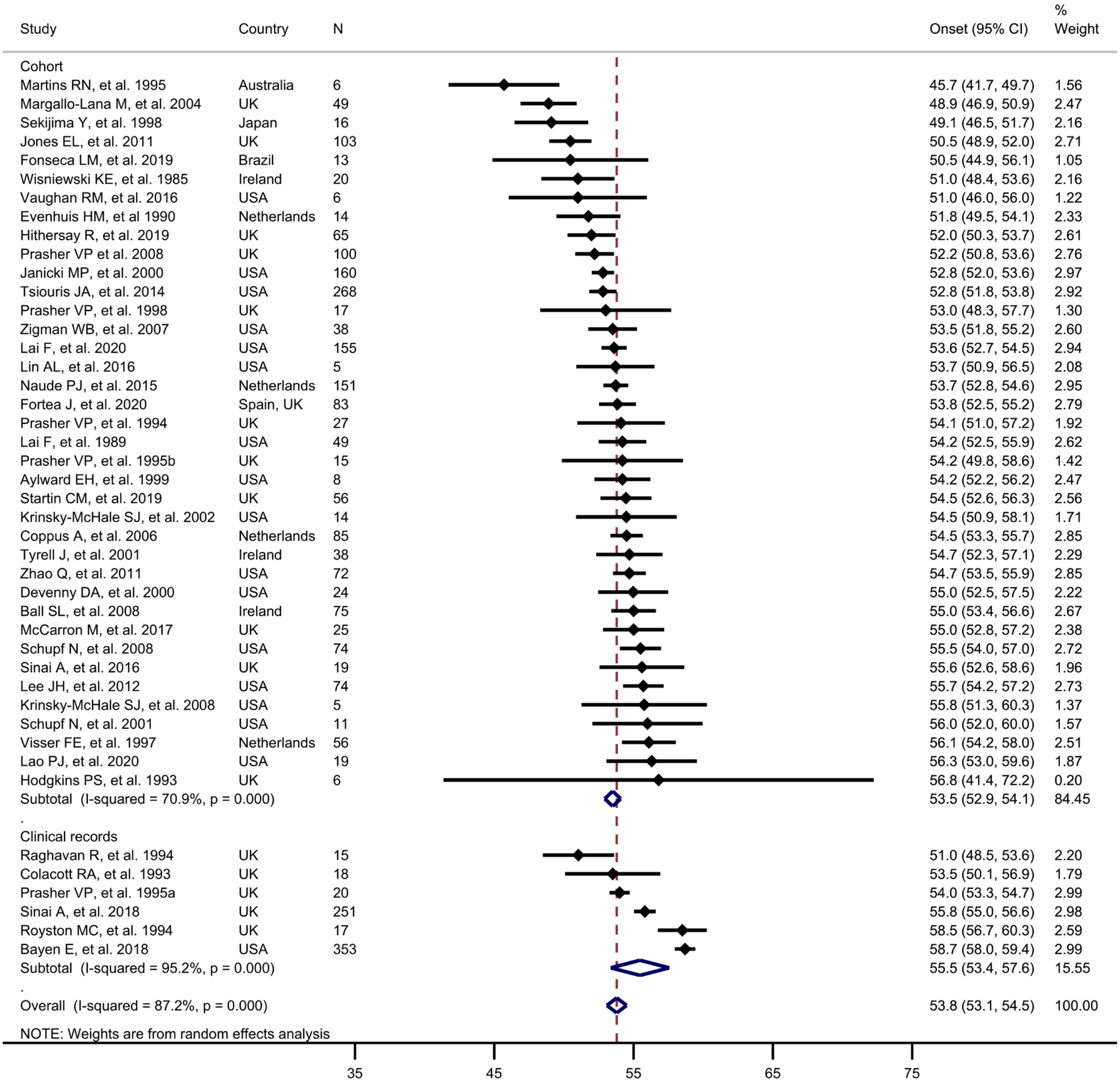

eFigure 5. Forest plot of mean age at onset or diagnosis (years) of Alzheimer disease dementia in adults with Down syndrome according to data source. The black diamond represents the mean with the arms reflecting the 95% confidence intervals (CIs). The unfilled blue diamond represents the overall pooled estimate, and its width represents the confidence interval.

© 2022 Iulita MF et al. *JAMA Network Open.*

eFigure 6. Association of Geographic Location and Age at Onset Estimate

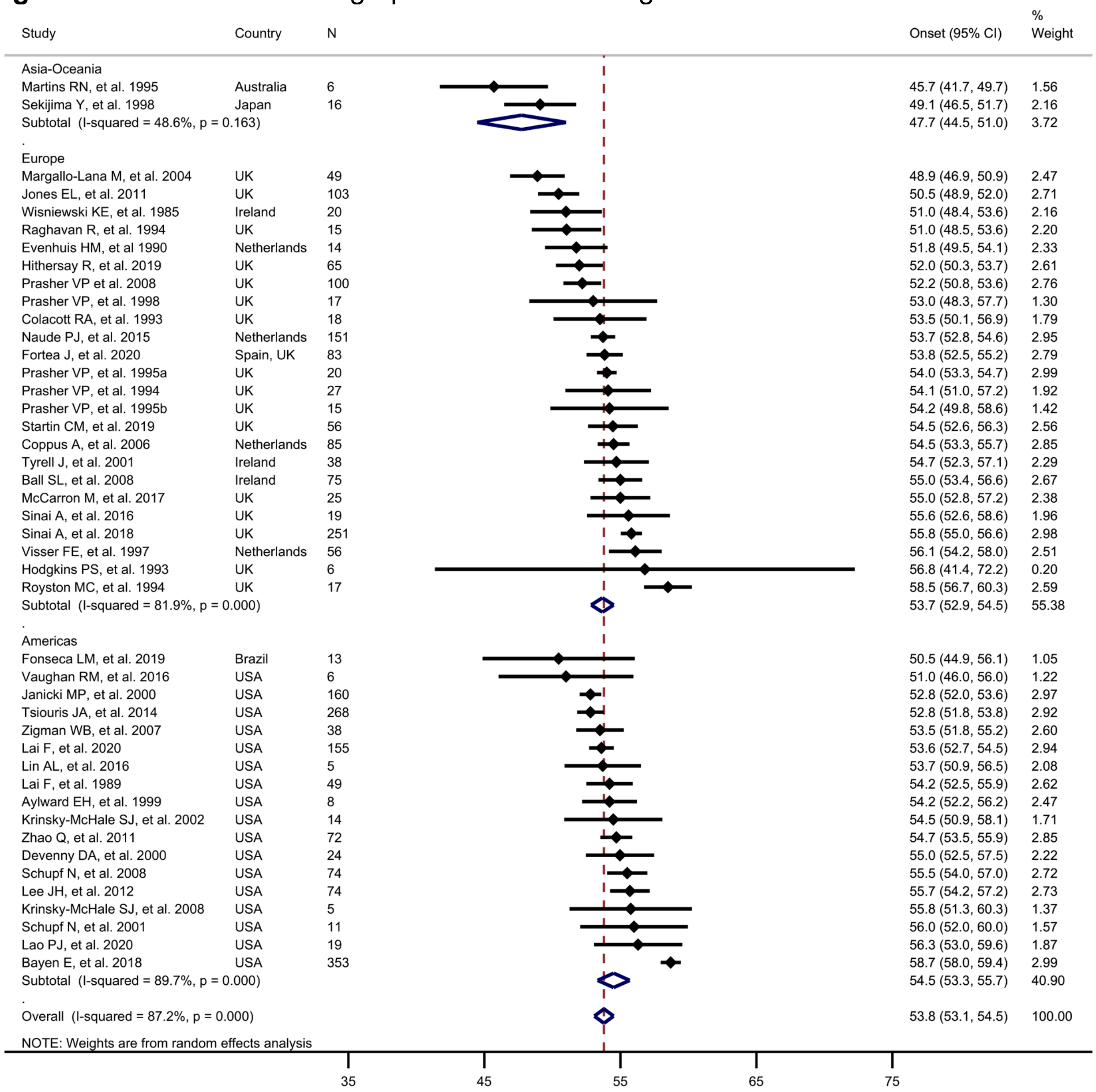

eFigure 6. Forest plot of mean age at onset or diagnosis (years) of Alzheimer disease dementia in adults with Down syndrome according to geographic location. The black diamond represents the mean with the arms reflecting the 95% confidence intervals (CIs). The unfilled blue diamond represents the overall pooled estimate, and its width represents the confidence interval.

© 2022 Iulita MF et al. *JAMA Network Open.*

eFigure 7. Association of Sample Size With Age at Onset Estimate

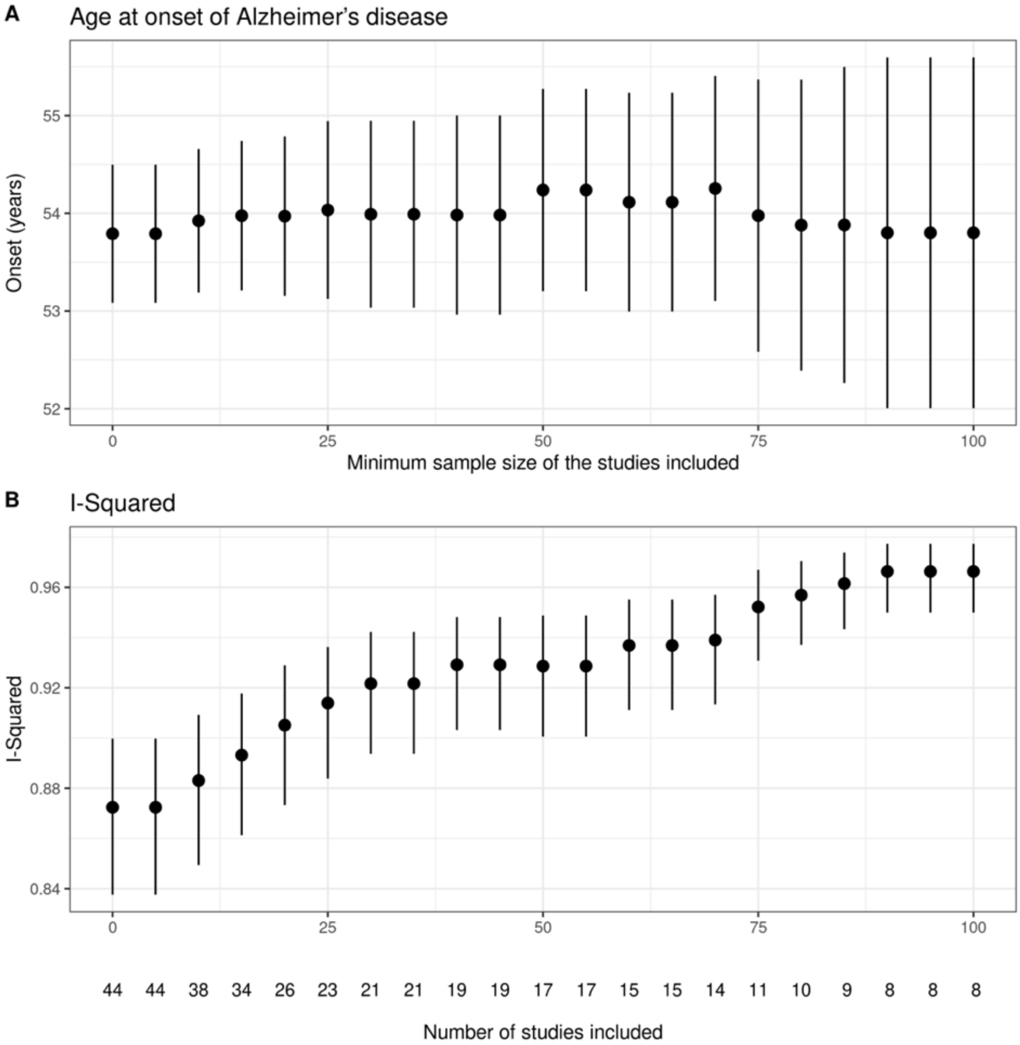

eFigure 7. Variation in the estimate of age at onset and heterogeneity (I^2) calculated in the meta-analysis according to sample size and number of studies.

© 2022 Iulita MF et al. *JAMA Network Open.*

eFigure 8. Variability in Age at Onset

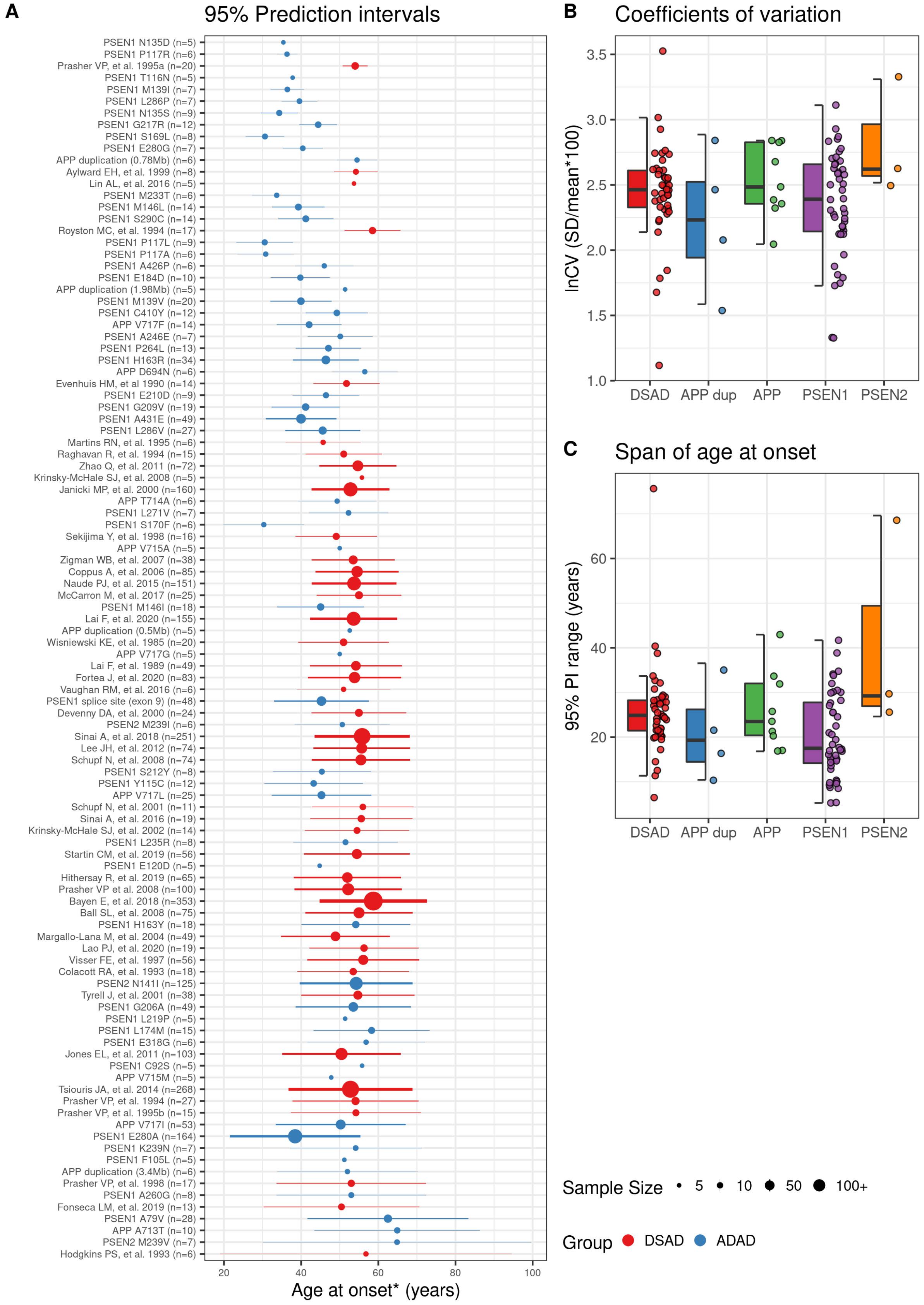

eFigure 8. A) 95% prediction intervals (PI) of individual studies on age at onset of Alzheimer disease in Down syndrome (DSAD) from our systematic review (44 studies), and from 60 studies on autosomal dominant Alzheimer disease (ADAD), as available in the systematic review of Ryman et al. 2014. B) Natural logarithm (Ln) of coefficients of variation (SD/mean*100) of the 44 included studies on DSAD, and from the 60 studies on ADAD separated by

© 2022 Iulita MF et al. *JAMA Network Open.*

pathogenic variant. C) The span of age at onset was calculated by computing the 95% PI range of the same studies included in (A) by subtracting the lower 95%PI to the upper 95%PI. A larger PI range indicates a wider interval (in years) in which 95% of individuals develop symptoms. Data is displayed in box and whiskers plot where the median is represented by the line and the lower and upper hinges correspond to the 25th and 75th percentiles. The upper whisker extends from the hinge to the largest value no further than 1.5*IQR (inter-quartile range) of the hinge. The lower whisker extends from the hinge to the smallest value no further than 1.5*IQR. The size of the dots in the distribution accounts for the sample size of the study. For all studies, sample size (N) was ≥5. APP dup = *APP* duplications; PSEN1 = *presenilin 1*; PSEN2: *presenilin 2*.

© 2022 Iulita MF et al. *JAMA Network Open.*

eFigure 9. Effect of Sample Size on Variability Estimates

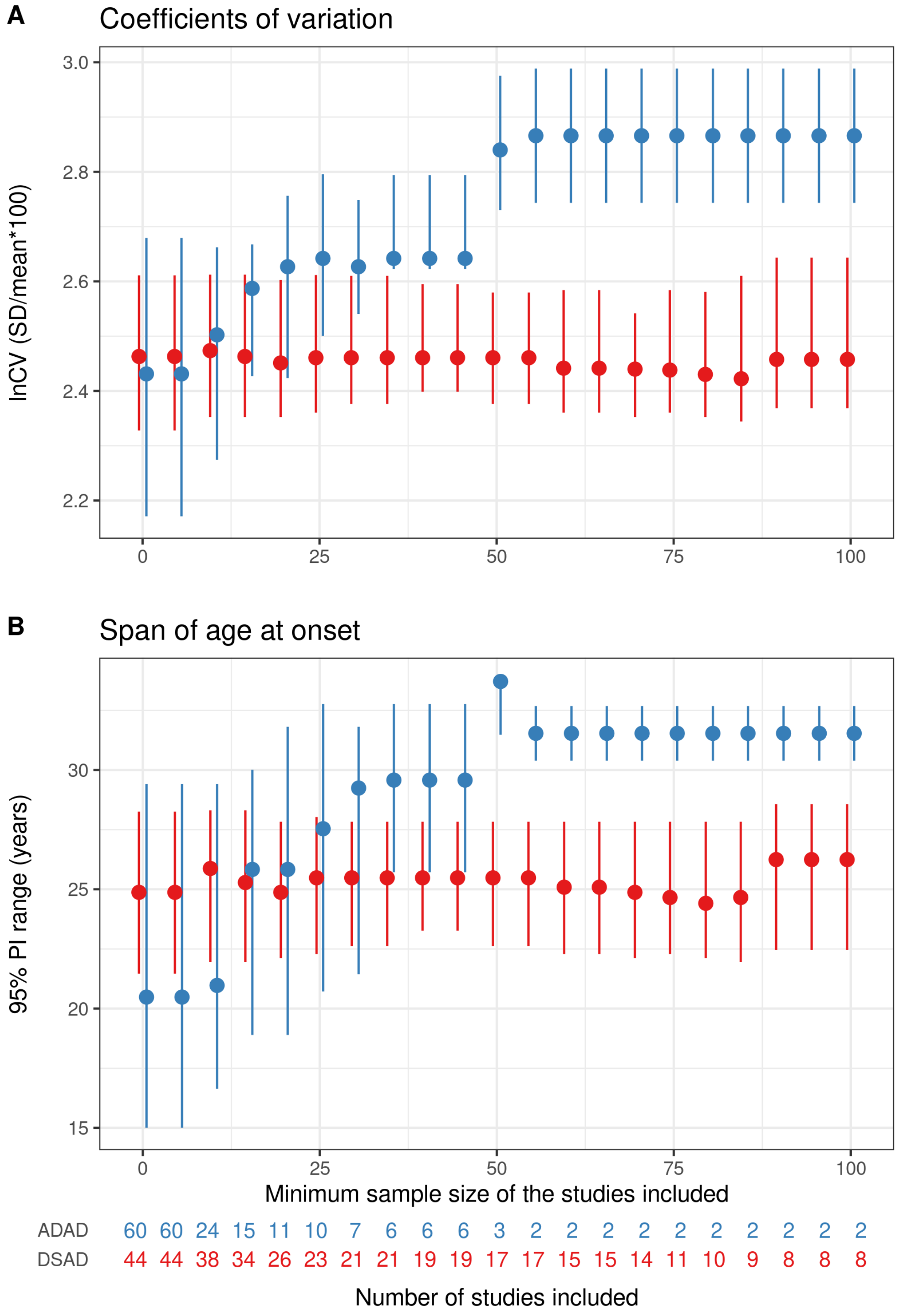

eFigure 9. A) Natural logarithm (Ln) of coefficients of variation (CV; SD/mean*100) and B) 95% prediction interval (PI) range of studies on age at onset of Alzheimer disease in Down syndrome (DSAD) from our systematic review (44 studies) and from the 60 studies on autosomal dominant Alzheimer disease (ADAD), as available in the systematic review of Ryman et al. 2014. In both cases, the minimal sample size for a study to be eligible for this analysis was ≥5. Representation of the change in CV and 95%PI range (y axis) with increasing minimal sample size of the studies included (x axis).

© 2022 Iulita MF et al. *JAMA Network Open.*

eFigure 10. Concordance Between Estimated and Real Distribution of Age at Death

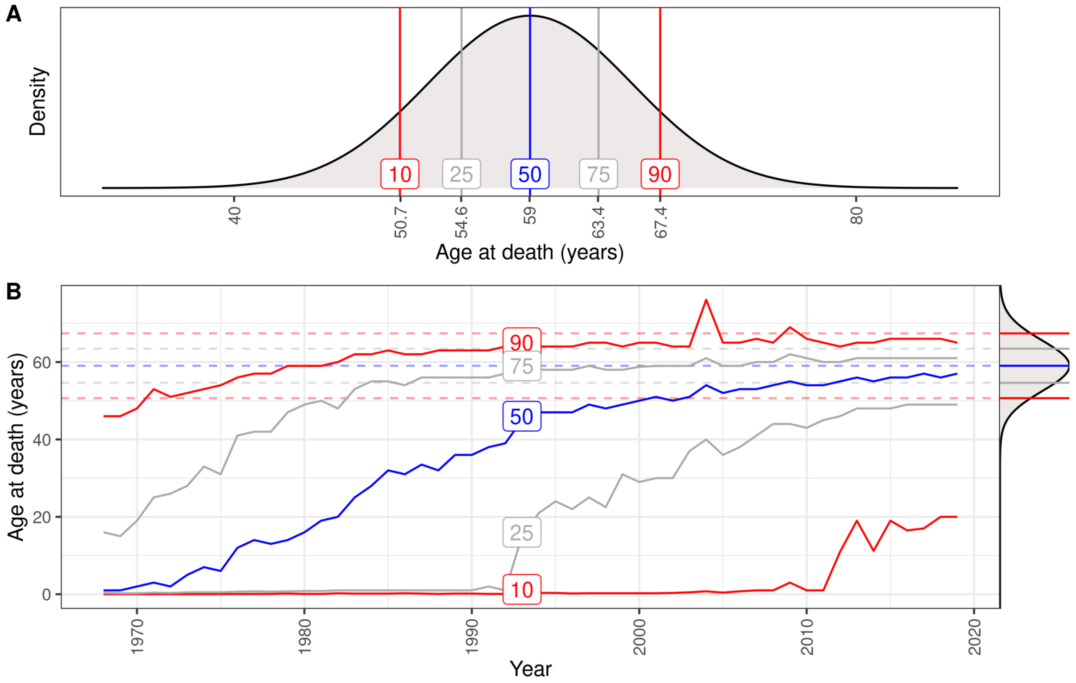

eFigure 10. A) Distribution of age at death in adults with Down syndrome based on the pooled estimate on age at death of Alzheimer disease calculated from the systematic review. B) Age at death in individuals with Down syndrome in the USA between 1968-2019. Red lines represent the 10th and 90th percentiles, gray lines represent the 25th and 75th percentiles and the median is depicted in blue. Top right: overlay of the predicted percentiles based on the distribution shown in (A).

© 2022 Iulita MF et al. *JAMA Network Open.*

eFigure 11. Distribution of Age at Death in Persons With Down Syndrome in the USA

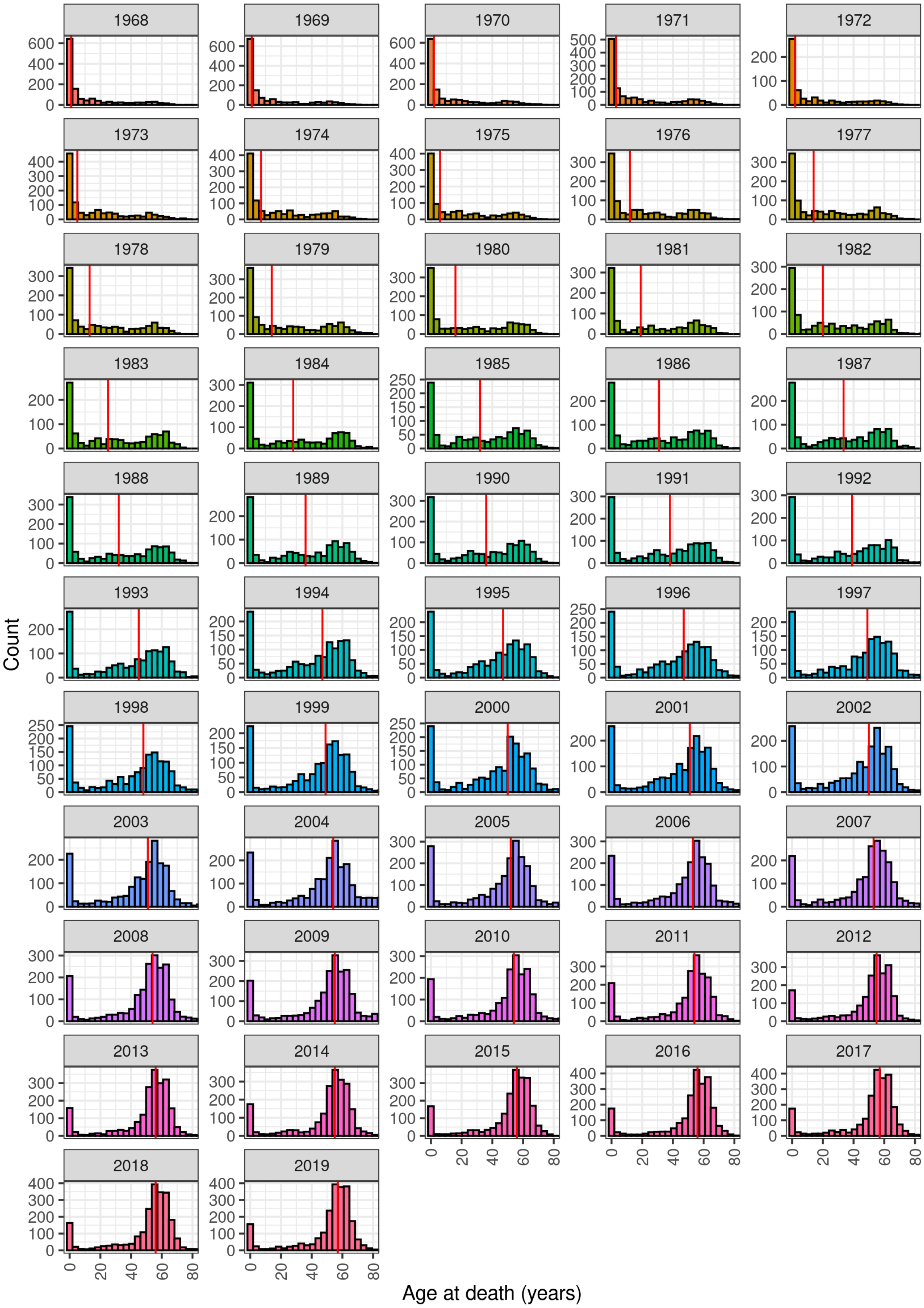

eFigure 11. Frequency distribution of age at death of individuals with Down syndrome between 1968-2019 obtained from US death certificates. Counts represent the number of deaths occurring each year in each age interval. The median is depicted by the red line. Note the dramatic increase in median age at death in the past decades from age 1 (1968) to age 57 (2019).

© 2022 Iulita MF et al. *JAMA Network Open.*

eFigure 12. Difference Between the Estimated and Observed Age at Death of People With Down Syndrome in the USA Between 1968-2018

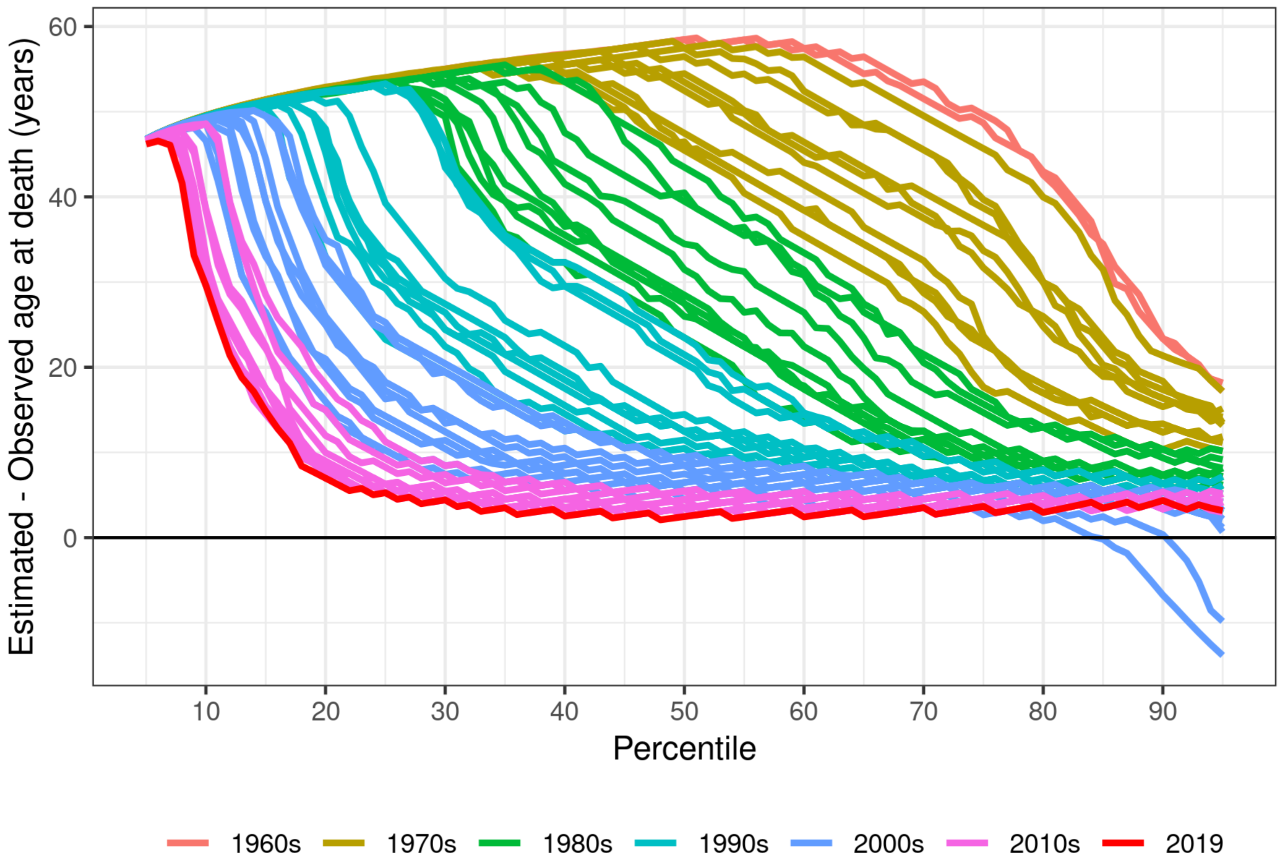

eFigure 12. Representation of the difference between the estimated age at death based on the hypothetical model of full Alzheimer disease penetrance (built with data from age at Alzheimer disease onset and duration) and the observed age at death obtained in US death certificates for each percentile and across each year.

© 2022 Iulita MF et al. *JAMA Network Open.*

eReferences.

1. McGrath J, Saha S, Welham J, El Saadi O, MacCauley C, Chant D. A systematic review of the incidence of schizophrenia: the distribution of rates and the influence of sex, urbanicity, migrant status and methodology. BMC Med 2004;2:13.

2. Higgins JPT, Thomas J, Chandler J, et al. Cochrane Handbook for Systematic Reviews of Interventions version 6.2 (updated February 2021). Cochrane, 2021 2021;Available from http://www.training.cochrane.org/handbook.

3. Fortea J, Vilaplana E, Carmona-Iragui M, et al. Clinical and biomarker changes of Alzheimer's disease in adults with Down syndrome: a cross-sectional study. Lancet 2020;395:1988-97.

© 2022 Iulita MF et al. *JAMA Network Open.*
